# Identification of the family of aquaporin genes and their expression in upland cotton (*Gossypium hirsutum *L.)

**DOI:** 10.1186/1471-2229-10-142

**Published:** 2010-07-13

**Authors:** Wonkeun Park, Brian E Scheffler, Philip J Bauer, B Todd Campbell

**Affiliations:** 1 USDA-ARS, Coastal Plains Soil, Water and Plant Research Center, 2611 West Lucas St., Florence, 29501, SC, USA; 2 USDA-ARS, MSA Genomics and Bioinformatics Research Unit, 141 Experiment Station Rd., Stoneville, 38776, MS, USA

## Abstract

**Background:**

Cotton (*Gossypium spp*.) is produced in over 30 countries and represents the most important natural fiber in the world. One of the primary factors affecting both the quantity and quality of cotton production is water. A major facilitator of water movement through cell membranes of cotton and other plants are the aquaporin proteins. Aquaporin proteins are present as diverse forms in plants, where they function as transport systems for water and other small molecules. The plant aquaporins belong to the large major intrinsic protein (MIP) family. In higher plants, they consist of five subfamilies including plasma membrane intrinsic proteins (PIP), tonoplast intrinsic proteins (TIP), NOD26-like intrinsic proteins (NIP), small basic intrinsic proteins (SIP), and the recently discovered X intrinsic proteins (XIP). Although a great deal is known about aquaporins in plants, very little is known in cotton.

**Results:**

From a molecular cloning effort, together with a bioinformatic homology search, 71 upland cotton (*G. hirsutum*) aquaporin genes were identified. The cotton aquaporins consist of 28 PIP and 23 TIP members with high sequence similarity. We also identified 12 NIP and 7 SIP members that showed more divergence. In addition, one XIP member was identified that formed a distinct 5^th ^subfamily. To explore the physiological roles of these aquaporin genes in cotton, expression analyses were performed for a select set of aquaporin genes from each subfamily using semi-quantitative reverse transcription (RT)-PCR. Our results suggest that many cotton aquaporin genes have high sequence similarity and diverse roles as evidenced by analysis of sequences and their expression.

**Conclusion:**

This study presents a comprehensive identification of 71 cotton aquaporin genes. Phylogenetic analysis of amino acid sequences divided the large and highly similar multi-gene family into the known 5 aquaporin subfamilies. Together with expression and bioinformatic analyses, our results support the idea that the genes identified in this study represent an important genetic resource providing potential targets to modify the water use properties of cotton.

## Background

Cotton is the most important naturally produced fiber in the world and represents a significant global agricultural commodity. Not taking into account additional economic value captured through cotton processing and associated byproducts, from 2005-2007, the average farm gate value of cotton equaled US $28 billion (World Bank, http://web.worldbank.org). Although the majority of cotton's value resides in the lint fiber used by textile manufacturers, additional benefits are obtained from cottonseed products that include animal feeds and various oil-derived products.

Similar to many other economically important crops, one of the major factors affecting both the quantity and quality of cotton production is water. Waddle [1] estimated that a successful cotton production system generally requires a minimum of 50 cm of water during the growing season. Hence, *in planta *efforts to decrease the quantity of water used and to improve cotton water use efficiency are highly desirable. At the molecular level, a potential target for manipulating water use efficiency is represented by the aquaporin proteins.

Aquaporin proteins represent a large family of the major intrinsic protein (MIP) superfamily and are known to facilitate transport of diverse small molecules including water and other small nutrients through biological membranes. Plant growth and development require water and nutrient uptake by transport mechanisms including a process mediated by aquaporins. Although the first plant aquaporin gene was cloned in soybean root nodules [2], it is now well known that plant aquaporins are ubiquitously distributed across plant tissue types. In higher plants, aquaporins consist of five subfamilies that include; 1) plasma membrane intrinsic proteins (PIP), 2) tonoplast intrinsic proteins (TIP), 3) NOD26-like intrinsic proteins (NIP), 4) small basic intrinsic proteins (SIP), and 5) the recently identified X (or unrecognized) intrinsic proteins (XIP) [3].

Aquaporin gene identification studies in plants have primarily relied on *in silico *methods. By using whole genome sequences, 35 aquaporin genes were identified in *Arabidopsis *[4], 33 from *Oryza sativa *L. [5], 28 from *Vitis vinifera *L. [6] and 23 from a moss, *Physcomitrella patens *[3]. Recently, 55 full-length aquaporins have been analyzed from *Populus trichocarpa *genome sequence data [7]. Expression sequence tag (EST) data analysis also identified the presence of at least 33 aquaporin genes from *Zea mays *L. [8]. In addition, many aquaporin isoforms have also been isolated from various plants including *Triticum aestivum *L. [9], *Nicotiana tabacum *L. [10], and *Pisum sativum *L. [11].

Although PIP, TIP, NIP and SIP subfamilies are conserved in plants, homology comparisons demonstrate that plant aquaporins have divergent sequence and function. For example, as a result of sequence divergence, the PIP subfamily has been classified further into two subgroups, PIP1 and PIP2. Aquaporins from each PIP subgroup can act individually in a different manner [12] or may interact together as a heterodimer to facilitate subcellular trafficking toward the plasma membrane [13,14]. In cotton, to date four PIP members have been characterized [15,16]. TIPs are divided phylogenetically into 5 different subgroups [4] and δ-TIP represented the first identified aquaporin gene in cotton [17]. The NIP proteins were initially thought to only be present in the nodules of nitrogen fixing legumes such as soybean [2,18]. However, NIP proteins have also been isolated from many non-leguminous plants including *Arabidopsis *[19], rice [5], and maize [20] and are known to represent a less conserved plant-specific aquaporin subfamily. Compared to PIP and TIP aquaporin subfamilies, the SIP aquaporin subfamily is not as well characterized. In *Arabidopsis, *SIP members appear to localize to the endoplasmic reticulum (ER) [21]. The XIP is a newly discovered, phylogenetically distinct subfamily and has been found widely in moss, fungi, and dicot plants [3]. To date, extensive functional characterization of the XIP subfamily has not been reported [7].

The role of aquaporins in plant water relations has been demonstrated structurally and physiologically [22,23]. As established earlier in human erythrocytes and bovine lens cells, the functional role of plant aquaporins is quite similar to that of animal aquaporins [24,25]. As a characteristic transmembrane channel protein, aquaporins have 6 membrane-spanning domains with two cytoplasmic termini. An additional important structural feature is the Asn-Pro-Ala (NPA) motif that is conserved in loops B (LB) and E (LE), in which two NPA motifs are placed in two short, fold-back alpha helices following second and fifth transmembrane helices, respectively. Along with aromatic/Arginine (ar/R) selective filters, this conserved NPA motif is known to provide substrate selectivity for molecular transport [26]. The expression of each aquaporin gene member is regulated differentially. Some aquaporins are constitutively expressed while many others are controlled in a tissue specific and environmentally sensitive manner [27,28]. In addition, proper subcellular localization represents another layer of mechanistic regulation of aquaporin activity that probably relies on its ability to form multimers between members of different subgroups [14].

While a great deal is known about aquaporin proteins and the genes that encode them in a wide variety of plant genera and species, very little is known about the aquaporins in cotton. Although the allotetraploid chromosome structure of upland cotton makes it an excellent model system to study polyploidization and genome duplication by mechanisms many crops have used to evolve from diploid ancestors [29,30], the complicated genetic structure of upland cotton challenges efforts aimed to discover gene families such as the aquaporins. Derived from two diploid species (AA and DD), the allopolyploid composition of the cotton genome affects gene expression primarily due to different contributions of alloalleles combined with allele-specific gene silencing [17,31]. In fact, several alloalleles of aquaporin genes have been described in cotton [17] and maize [32,33], although no allele-specific gene expression analysis has been described for aquaporins. Thus, it is interesting to reveal how the evolutionally conserved, multigene aquaporin family behaves in polyploid species such as cotton. To our knowledge there have only been seven aquaporin genes reported from allotetraploid cotton in the literature [15-17]. Therefore, a first step in investigating the role of aquaporins in cotton water relations is to identify the aquaporin gene family. With that in mind, our objective in this study was to identify cotton aquaporin genes and investigate both their structural properties and expression patterns.

## Results

### Isolation of the aquaporin gene family from upland cotton

Using PCR cloning and EST data, we identified 71 candidate cotton aquaporin genes. Information including gene names, accession numbers, identification methods, the length of deduced polypeptides, and gene homology is summarized in Table 1. Twenty-five genes were identified by both PCR and EST homology searches. Twenty genes were detected by PCR cloning alone (See Additional file 1 for the sequence information of primers used in PCR cloning) and 26 genes were identified using only bioinformatic database search methods. Eighteen out of 45 PCR-generated clones were isolated repeatedly up to ten times while 27 PCR clones were identified once. Among these 27 PCR clones, 11 were also found using bioinformatic database search methods. RACE PCR was also used to clone the full-length sequence of *PIP1;11*, *TIP1;7*, *TIP2;3*, and *XIP1;1*.

**Table 1:** Summary of cotton aquaporin genes

Name	**Accession Number**^**a **^**or PUT ID**^**†**^	Length (amino acid)	**Highest similarity (%)**^**b**^		Identification		Reference
				
			Cotton	Other plants	**Method**^**c**^	**Number of clone**^**d**^	
***PIPs***							
*PIP1;1*	ABK60194	289	PIP1;14(100)	ACL14797(94)^e^	1, 2, 4	8/4	Li et al., 2009
*PIP1;2*	ABR68794	287	PIP1;13(97)	EEF51202(96)^f^	1, 4	1/1	Liu et al., 2008
*PIP1;3*	ABD63904	287	PIP1;15(99)	EEF51202(96)^f^	1, 3	1/1	
*PIP1;4*	BK007045	287	PIP1;15(99)	EEF51202(95)^f^	2, 3	1/1	
*PIP1;5*	BK007046	278	PIP1;12(95)	EEF51202(96)^f^	3		
*PIP1;6*	BK007047	287	PIP1;1(87)	EEF05326(95)^e^	3		
*PIP1;7*	BK007048	288	PIP1;10 (98)	ACL14797(94)^e^	3		
*PIP1;8*	BK007049	259	PIP1;9(92)	ABK95101(92)^e^	3		
*PIP1;9*	BK007050	257	PIP1;8(92)	ACL14797(92)^e^	3		
*PIP1;10*^‡^	GU998827	177	PIP1;7(98)	ACF39902(95)^g^	1	1/1	
*PIP1;11*	GU998828	289	PIP1;14(99)	ACL14797(94)^e^	1, 2	9/4	
*PIP1;12*^‡^	GU998829	267	PIP1;4(98)	EEF51202(95)^f^	1	4/3	
*PIP1;13*^‡^	GU998830	266	PIP1;3(100)	EEF51202(96)^h^	1	1/1	
*PIP1;14*	PUT41616	289	PIP1;1 (100)	ACL14797(94)^e^	3		
*PIP1;15*	PUT20977	287	PIP1;3(99)	EEF51202(96)^f^	3		
*PIP2;1*	ABK60195	285	PIP2;3(94)	EEF42954(93)^f^	4		Li et al., 2009
*PIP2;2*	ABK60196	286	PIP2;12(100)	EEF42953(93)^f^	4		Li et al., 2009
*PIP2;3*	PUT368101081	285	PIP2;13(99)	ABK96511(93)^e^	1, 3	1/1	
*PIP2;4*	BK007051	270	PIP2;11(100)	ABN14351(92)^h^	2, 3	1/1	
*PIP2;5*	PUT818101073	278	PIP2;7(100)	ABN14351(96)^h^	3		
*PIP2;6*	PUT818101073	278	PIP2;5(99)	ABN14351(96)^h^	1, 2, 3	8/5	
*PIP2;7*	PUT58401	261	PIP2;5(100)	ABN14351(96)^h^	3		
*PIP2;8*	PUT472101080	280	PIP2;4(83)	AtPIP2;7(89)^i^	3		
*PIP2;9*	ACB42441	278	PIP2;11(99)	ABK95359(94)^e^	1, 2, 3	6/4	
*PIP2;10*	BK007052	271	PIP2;13(80)	EEF32087(87)^f^	3		
*PIP2;11*	ACB42440	278	PIP2;4(100)	ABN14351(95)^h^	3		
*PIP2;12*	PUT51785	286	PIP2;2(100)	EEF42953(93)^f^	3		
*PIP2;13*	PUT368101081	285	PIP2;3(99)	EEF42953(94)^f^	3		
***TIPs***							
*TIP1;1*	ACP28878	251	*TIP*1;3(100)	ACI95283(95)^j^	2, 3, 4	1/1	Li et al., 2009
*TIP1;2*	ABR68795	252	TIP1;7(98)	EEF31283(95)^f^	1, 4	6/1	Liu et al., 2008
*TIP1;3*	BK007053	251	TIP1;1(100)	ACI95283(95)^j^	2, 3	2/1	
*TIP1;4*	BK007054	251	TIP1;6(99)	ACI95283(93)^j^	1, 3	6/4	
*TIP1;5*	BK007055	249	TIP1;14(99)	ACI95283(92)^j^	3		
*TIP1;6*^‡^	BK007056	284	TIP1;4(98)	ACI95283(93)^j^	1, 3	4/2	
*TIP1;7*	GU998831	252	TIP1;2(98)	EEF31283(95)^f^	1, 3	6/2	
*TIP1;8*	BK007057	251	TIP1;1(99)	ACI95283(94)^j^	3		
*TIP1;9*^‡^	GU998832	179	TIP1;13(98)	EEF31283(95)^f^	1	1/1	
*TIP1;10*^‡^	GU998833	179	TIP1;13(98)	EEF31283(96)^f^	1	1/1	
*TIP1;11*^‡^	GU998834	179	TIP1;10(93)	EEF31283(96)^f^	1	2/2	
*TIP1;12*^‡^	GU998835	179	TIP1;7 (98)	AAW02943(95)^h^	1	1/1	
*TIP1;13*^‡^	GU998836	179	TIP1;2(99)	AAW02943(96)^h^	1	1/1	
*TIP1;14*	PUT83401	249	TIP1;5(99)	ACI95283(92)^j^	1, 2, 3	10/5	
*TIP2;1*	AAB04557	248	TIP2;3(99)	EEF46419(94)^f^	1, 3, 4	1/1	Ferguson et al., 1997
*TIP2;2*^‡^	BK007058	245	TIP2;5(99)	EEF46419(93)^f^	2, 3	1/1	
*TIP2;3*	GU998837	248	TIP2;1(99)	EEF46419(94)^f^	1, 2, 3	4/3	
*TIP2;4*^‡^	GU998838	146	TIP2;5(94)	EEE83038(94)^e^	2	1/1	
*TIP2;5*^‡^	GU998839	146	TIP2;4(94)	EEE83038(94)^e^	1	1/1	
*TIP2;6*	PUT96185	250	TIP2;7(99)	CAO23095(96)^h^	3		
*TIP2;7*	PUT96185	250	TIP2;6(99)	CAO23095(95)^h^	3		
*TIP4;1*	BK007059	246	TIP4;2(100)	EEE93071(92)^e^	1, 3	3/1	
*TIP4;2*	BK007060	246	TIP4;1(100)	EEE93071(92)^e^	3		
***NIPs***							
*NIP1;1*	BK007061	280	NIP1;3(98)	EEF40132(88)^f^	1, 2, 3	5/3	
*NIP1;2*^‡^	GU998840	170	NIP1;3(99)	AAS48064(92)^k^	1, 2	4/3	
*NIP1;3*^‡^	GU998841	174	NIP1;2(99)	AAS48064(93)^k^	1	1/1	
*NIP2;1*	PUT77848	259	NIP1;2(50)	EEF27965(86)^f^	3		
*NIP5;1*^‡^	PUT43349	256	NIP5;2(97)	EEF43506(94)^f^	3		
*NIP5;2*^‡^	PUT43348	221	NIP5;1(97)	EEF43506(94)^f^	3		
*NIP6;1*^‡^	BK007062	280	NIP6;6(100)	EEE82602(92)^e^	1, 2, 3	10/5	
*NIP6;2*^‡^	PUT83990	288	NIP6;5(99)	EEF35060(90)^f^	2, 3	1/1	
*NIP6;3*^‡^	GU998842	140	NIP6;4(98)	EEE79702(92)^e^	2	1/1	
*NIP6;4*^‡^	GU998843	140	NIP6;3(98)	EEE79702(92)^e^	2	1/1	
*NIP6;5*^‡^	GU998844	203	NIP6;6(99)	EEE79702(94)^e^	2	1/1	
*NIP6;6*^‡^	GU998845	234	NIP6;1(100)	EEE79702(93)^e^	1	1/1	
***SIPs***							
*SIP1;1*	PUT7068	240	SIP1;2(73)	ABD46741(77)^h^	3		
*SIP1;2*	BK007063	247	SIP1;3(73)	EEE99776(84)^e^	3		
*SIP1;3*	BK007064	241	SIP1;5(98)	EEE37542(75)^f^	1, 3	1/1	
*SIP1;4*	PUT22448	243	SIP1;7(98)	ACU20408(71)^l^	1, 3	1/1	
*SIP1;5*^‡^	GU998846	198	SIP1;3(98)	EEE37542(71)^f^	1	1/1	
*SIP1;6*^‡^	GU998847	198	SIP1;5(92)	EEE37542(71)^f^	2	1/1	
*SIP1;7*^‡^	GU998848	198	SIP1;4(97)	ACU24419(74)^l^	1	5/3	
***XIP***							
*XIP1;1*	GU998849	302		EEE86940(86)^e^	1, 3	9/3	

^†^: GenBank accessions from GU998827-GU998849 are submitted in this study. Also, nucleotide sequence data reported are available in the Third Party Annotation Section of the DDBJ/EMBL/GenBank databases under the accession numbers TPA: BK007045-BK007064. PlantGDB-assembled Unique Transcript (PUT) ID numbers are provided as sources of indicated genes.

^‡^: Partial sequence.

^a^: Accession number from NCBI.

^b^: A gene that shows highest identity in cotton by ClustalW or highest similarity in other plants by BLASTP. Parenthesis indicates percentage of identity at amino acid level.

^c^: 1; Amplification of cDNA, 2; Amplification of genomic DNA, 3; EST BLAST, 4; Published literature

^d^: Number of clones isolated/Number of independent experiments.

^e^: *Populus*, ^f^: Common bean, ^g^: *Camellia*, ^h^: Grape, ^i^: *Arabidopsis*, ^j^: *Sinapis*, ^k^: *Medicago*, ^l^: Soybean.

**Figure 1:**
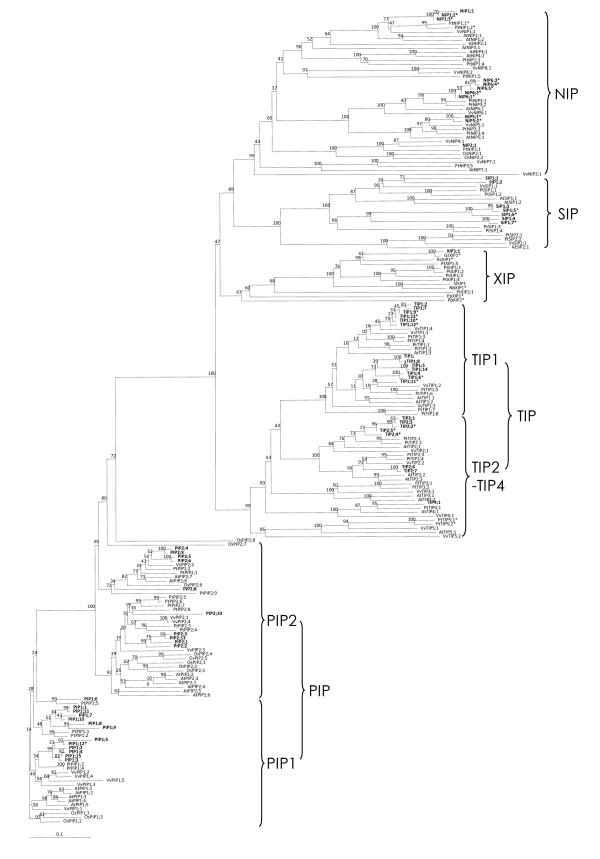
**Phylogenetic analysis of 71 members of the cotton aquaporin family with members of other plants**. Deduced amino acid sequences were aligned using ClustalW2 and the phylogenetic tree was generated using Bootstrap N-J tree (1,000 resamplings) method and TreeView program (v1.6.6). The name of each group and subgroup is indicated next to the corresponding group. The distance scale denotes the number of amino acid substitutions per site. Gr, *Gossypium raimondii*; At, *Arabidopsis thaliana*; Vv, *Vitis vinifera*; Os, *Oryza sativa*; Pp, *Physcomitrella patens*; Pt; *Populus trichocarpa*; Rc, *Ricinus communis*; Nb, *Nicotiana benthamiana*; Sl, *Solanum lycopersicum*. Bold gene names with no species initials are for cotton aquaporins. Asterisks denote genes with partial sequence. Only 63 cotton aquaporins were included here among those identified in this study because eight of the aquaporin transcripts encode proteins of identical sequence resulting in only 63 unique protein sequences.

By comparing amino acid sequences of cotton aquaporins with previously identified plant aquaporins, cotton aquaporin candidates were successfully classified as 28 PIPs (15 PIP1s and 13 PIP2s), 23 TIPs (14 TIP1s and 9 other TIPs), 12 NIPs, 7 SIPs and 1 XIP (Figure 1). Eight of the deduced amino acid sequences (PIP1;13, PIP1;14, PIP2;11, PIP2;12, PIP2;7, TIP1;3, TIP4;2 and NIP6;6) encoded 100% identical sequences to other members (Additional file 2), and 63 cotton aquaporin protein sequences (encoded from 71 genes) were phylogenetically analyzed with members from other plants (Figure 1). As a rule, in naming the identified cotton aquaporin genes, we followed previous nomenclature of other plants guided by sequence homology and phylogenetic analysis. To systematically classify cotton aquaporin genes and determine phylogenetic relationships with aquaporin genes from other plants, full-length aquaporin members from *Arabidopsis*, rice, grape and *Populus *were included in phylogenetic analysis and the result is presented in Figure 1. For XIP aquaporin comparisons, several XIPs were also added from moss (*P. patens*), tomato, tobacco, and common bean [3].

Overall, cotton aquaporin genes followed the phylogenetic clustering pattern as previously reported in other plants [4,7]. PIP subfamily members were further divided into PIP1 and PIP2 subgroups and TIPs were partitioned into TIP1 and two other TIP subgroups (TIP2 and TIP4). Similar to *Populus *aquaporins, many cotton PIP and TIP members had a tendency to cluster with other members in the same subfamily. Four and one subgroups appeared for NIP (NIP1, NIP2, NIP5, and NIP6) and SIP (SIP1), respectively. We did not identify cotton members of several subgroups identified in other plants; these subgroups included TIP3, NIP3, and NIP4. In Table 2, sequence information is summarized to show conserved amino acid residues, the prediction of transmembrane domains, and subcellular localization.

**Table 2:** Conserved amino acid residues and the prediction of transmembrane domains and subcellular localization

Name	Ar/R selectivity filter				**NPA**^**a **^**(LB/LE)**	Froger's Position (P1 - P5)					**TMH**^**b**^	Subcellular localization
					
	H2	H5	LE1	LE2		P1	P2	P3	P4	P5		
PIP1;1	F	H	T	R	NPA/NPA	M	S	A	F	W	6	PM^c^
PIP1;2	F	H	T	R	NPA/NPA	Q	S	A	F	W	6	PM^c^
PIP1;3	F	H	T	R	NPA/NPA	E	S	A	F	W	6	PM^c^
PIP1;4	F	H	T	R	NPA/NPA	E	S	A	F	W	6	PM^c^
PIP1;5	F	H	T	R	NPA/NPA	Q	S	A	F	W	5	PM^c^
PIP1;6	F	H	T	R	NPA/NPA	Q	S	A	F	W	5	PM^c^
PIP1;7	F	H	T	R	NPA/NPA	M	S	A	F	W	5	PM^c^
PIP1;8	F	H	T	R	NPA/NPA	M	S	A	-	-	5	PM^c^
PIP1;9	F	H	T	R	NPA/NPA	M	S	A	-	-	5	PM^c^
PIP1;10^†^	-	H	T	R	NPA/NPA	M	S	A	F	-	-	-
PIP1;11	F	H	T	R	NPA/NPA	M	S	A	F	W	6	PM^c^
PIP1;12^†^	F	H	T	R	NPA/NPA	E	S	A	F	W	5	PM^c^
PIP1;13^†^	F	H	T	R	NPA/NPA	E	S	A	F	W	5	PM^c^
PIP1;14	F	H	T	R	NPA/NPA	M	S	A	F	W	6	PM^c^
PIP1;15	F	H	T	R	NPA/NPA	E	S	A	F	W	6	PM^c^
												
PIP2;1	F	H	T	R	NPA/NPA	Q	S	A	F	W	6	PM^c^
PIP2;2	F	H	T	R	NPA/NPA	Q	S	A	F	W	6	PM^c^
PIP2;3	F	H	T	R	NPA/NPA	Q	S	A	F	W	6	PM^c^
PIP2;4	F	H	T	R	NPA/NPA	M	S	A	F	W	6	PM^c^
PIP2;5	F	H	T	R	NPA/NPA	M	S	A	F	W	6	PM^c^
PIP2;6	F	H	T	R	NPA/NPA	M	S	A	F	W	6	PM^c^
PIP2;7	F	H	T	R	NPA/NPA	M	S	A	F	W	6	PM^c^
PIP2;8	V	H	T	R	NPA/NPA	M	S	A	F	W	6	PM^c^
PIP2;9	F	H	T	R	NPA/NPA	M	S	A	F	W	6	PM^c^
PIP2;10	F	H	T	R	NPA/NPA	Q	S	A			5	PM^c^
PIP2;11	F	H	T	R	NPA/NPA	Q	S	A	F	W	6	PM^c^
PIP2;12	F	H	T	R	NPA/NPA	M	S	A	F	W	6	PM^c^
PIP2;13	F	H	T	R	NPA/NPA	Q	S	A	F	W	6	PM^c^
												
TIP1;1	H	I	A	V	NPA/NPA	T	S	A	Y	W	6	V^c^
TIP1;2	H	I	A	V	NPA/NPA	T	S	A	Y	W	6	V^c^
TIP1;3	H	I	A	V	NPA/NPA	T	S	A	Y	W	6	V^c^
TIP1;4	H	I	A	V	NPA/NPA	T	S	A	Y	W	6	
TIP1;5	H	I	A	V	NPA/NPA	T	S	A	Y	W	7	V^c^
TIP1;6^†^	H	I	A	V	NPA/NPA	T	S	A	Y	W	6	-
TIP1;7	H	I	A	V	NPA/NPA	T	S	A	Y	W	6	
TIP1;8	H	I	A	V	NPA/NPA	T	S	A	Y	W	7	V^c^
TIP1;9^†^	H	I	A	V	NPA/NPA	T	S	A	-	-	-	-
TIP1;10^†^	H	I	A	V	NPA/NPA	T	S	A	-	-	-	-
TIP1;11^†^	H	I	A	V	NPA/NPA	T	S	A	-	-	-	-
TIP1;12^†^	H	I	A	V	NPA/NPA	T	S	A	-	-	-	-
TIP1;13^†^	H	I	A	V	NPA/NPA	T	S	A	-	-	-	-
TIP1;14	H	I	A	V	NPA/NPA	T	S	A	Y	W	7	V^c^
												
TIP2;1	H	I	G	R	NPA/NPA	T	S	A	Y	W	7	PM/V^c^
TIP2;2	H	I	G	R	NPA/NPA	T	S	A	Y	W	6	V^c^
TIP2;3^†^	H	I	G	R	NPA/NPA	T	S	A	Y	W	7	-
TIP2;4	-	I	G	R	NPA/NPA	T	S	A	Y	W	-	V^c^
TIP2;5^†^	-	I	G	R	-/-	T	S	A	Y	W		-
TIP2;6	H	I	G	R	NPA/NPA	T	S	A	Y	W	6	V^c^
TIP2;7	H	I	G	R	NPA/NPA	T	S	A	Y	W	6	V^c^
TIP4;1	H	I	A	R	NPA/NPA	T	S	A	Y	W	6	PM^c^
TIP4;2	H	I	A	R	NPA/NPA	T	S	A	Y	W	6	PM^c^
												
NIP1;1	W	V	A	R	NPA/NPA	F	S	A	Y	L	6	PM^c^
NIP1;2^†^	W	V	A	-	NPA/NPA	F	-	-	-	-	-	-
NIP1;3^†^	W	V	A	R	NPA/NPA	F	S	-	-	-	-	-
NIP2;1	G	S	G	R	NPA/NPA	L	S	A	Y	V	5	PM^c^
NIP5;1^†^	A	I	G	R	NP***S***/NP***V***	F	T	A	Y	L	-	-
NIP5;2^†^	A	I	G	R	NP***S***/NP***V***	F	T	A	Y	L	-	-
												
NIP6;1^†^	T	I	A	R	NPA/NP***V***	F	T	A	Y	F	6	-
NIP6;2^†^	T	I	A	R	NPA/NP***V***	F	T	A	Y	F	6	-
NIP6;3^†^	T	-	-	-	NP***T***/-	-	-	-	-	-	-	-
NIP6;4^†^	T	-	-	-	NP***T***/-	-	-	-	-	-	-	-
NIP6;5^†^	T	-	-	-	NPA/-	F	-	-	-	-	-	-
NIP6;6^†^	T	I	A	-	NPA/NP***V***	F	-	-	-	-	-	-
												
SIP1;1	F	I	P	F	***D***PA/NPA	I	A	A	Y	W	5	PM/G^c^,ER^e^, S^d^
SIP1;2	I	T	P	N	NP***T***/NPA	M	A	A	Y	W	5	ER^e^, S^d^
SIP1;3	V	T	P	N	NP***T***/NPA	L	A	A	Y	W	5	ER^e^
SIP1;4	V	T	A	S	NPA/NPA	I	A	A	Y	W	3	V^c^, ER^e^
SIP1;5^†^	V	T	P	N	NP***T***/NPA	L	A	A	-	-	-	-
SIP1;6^†^	V	T	P	N	NP***T***/NPA	L	A	A	-	-	-	-
SIP1;7^†^	V	T	A	R	NPA/NPA	I	A	A	-	-	-	-
												
XIP1;1	I	T	V	R	NP***V***/NPA	V	C	A	F	W	7	PM/V^c^

^†^: Partial sequence.

^a^: Bold italic letters denote unusual amino acids in NPA motifs.

^b^: Number of transmembrane helices (TMH) predicted by TMHMM analysis tool [64].

^c^: PSORT (PM, plasma membrane; C, cytoplasm; V, vacuole; G, golgi) [65].

^d^:TargetP (S, secretion)[66].

^e^: Prodotar (E, endoplasmic reticulum) http://urgi.versailles.inra.fr/predotar/predotar.html.

-: Not determined due to limited sequence information.

By analyzing 197 total aquaporin genes from cotton and several different plant species, many aquaporin members from allotetraploid cotton appeared phylogenetically more close to members from *Populus *and grape for PIP and TIP subfamilies. The close relationship between cotton and *Populus *aquaporins was less apparent for NIP, SIP, and XIP subfamilies.

### Cotton aquaporin gene family

#### PIP

PIP1 open reading frames (ORFs) of full-length clones were predicted to encode polypeptides of 257 - 289 amino acids in length with 82 - 100% sequence identity in cotton. It has been shown that in many crops including cotton, there are duplicated copies of genes resulting from genome merger and/or genome doubling [34,35]. In the present study, we identified several aquaporin genes with very high sequence similarity sharing 99 -100% identity in predicted amino acid sequences. These candidates include 'PIP1;1, PIP1;11, and PIP1;14', and 'PIP1;3, PIP1;4, PIP1;13 and PIP1;15' (Additional file 2). More detailed information for these genes including expression analysis is summarized in Table 3. The length of the PIP2 ORF ranged from 261 to 286 amino acids with 71 - 100% identity. PIP2;2, PIP2;4, and PIP2;5 are 100% identical to PIP2;12, PIP2;11, and PIP2;7, respectively. In addition, PIP2;3 and PIP2;13 shared 99% amino acid sequence identity. PIP1 and PIP2 groups showed 63 - 81% identity with each other. Structurally, PIP1 had a longer extension at the N-terminus, while PIP2 had a longer C-terminal end (Additional file 3). However, as shown in Table 1, the overall lengths of estimated ORFs were quite similar between PIP1 and PIP2. All of the PIP members contained conserved dual NPA motifs in loops B and E, respectively; whereas the aromatic/Arg (ar/R) selectivity filter harbored identical residues, Phe (F), His (H), Thr (T), and Arg (R) in all of the PIP members except PIP2;8 where Phe (F) was replaced by Val (V). In addition, four out of five (P1 to P5) Froger's positions, recognized as differentially conserved residues between aquaporins and aquaglyceroporins and thereby providing functional specificities, were conserved in all of the full-length PIP sequences. Only the P1 site was less conserved, as one of three amino acids Glu (E), Met (M), and Gln (Q) appeared in this position (Table 2). In addition to dual NPA motifs, the ar/R selectivity filter, and five Froger's positions, it was reported that Val (V) for PIP2 and Ile (I) for PIP1 residues near the second NPA motif have been identified as key residues for water channel activity in radish [36]. The Val (V) and Ile (I) residues were also conserved in cotton PIP1 and PIP2 members, respectively.

**Table 3:** Analysis of cotton aquaporin sequence contigs in PlantGDB-assembled unique transcripts (PUT)^a^

PUT ID	Gene	Sequence difference in ORF (nt/aa)	No. EST of gene/No. EST of PUT	**Tissue abundance**^**b**^	**RT-PCR detection in Figure 3**^**c**^
41616	PIP1;1^†^	4/0	16/18	Fiber (10/16)	Co-detected
	PIP1;14		2/18	Stem (2/2)	Co-detected
					
					
20977	PIP1;3^†^	6/2	8/10	Fiber (7/8)	Detected
	PIP1;15		2/10	Mixed	n.a.
					
					
51785	PIP2;2	9/0	5/9	Mixed	n.a.
	PIP2;12		4/9	Mixed	n.a.
					
					
368101081	PIP2;3^†^	9/3	9/13	Stem (5/9)	n.a.
	PIP2;13		4/13	Stem (2/4)	n.a.
					
818101073	PIP2;5	9/3	> 20/92	Fiber (> 20)	n.a.
	PIP2;6^†^		> 20/92	Fiber (> 20)	n.a.
					
					
295101081	TIP1;1^†^	15/2^‡^	16/50	Stem (9/16)	n.a.
	TIP1;3^†^		> 20/50	Immature ovule (19/> 20)	n.a.
	TIP1;8		1/50	Fiber (1/1)	Detected
					
83401	TIP1;5	16/3	25/29	Fiber (18/25)	n.a.
	TIP1;14		4/29	Ovule (3/4)	n.a.
					
					
3730	TIP2;1^†^	8/1	16/28	Mixed	n.a.
	TIP2;3^†^		12/28	Stem (5), root (2)	Detected
					
					
96185	TIP2;6	10/2	2/7	Root (2/2)	Co-detected
	TIP2;7		5/7	Mixed	Co-detected
					
					
83990	NIP6;1^†^	2/1	1	Root (1/1)	n.a.
	NIP6;2^†^		1	Fiber (1/1)	Detected

^†^: Genes also identified by PCR cloning.

^‡^: Overall difference among three sequences.

^a^: Manual inspection was performed to discriminate a set of genes that originally belonged to a single PUT sequence as indicated.

^b^: mRNA abundance in specific tissues was determined from PlantGDB EST information.

^c^: RT-PCR primers used in Figure 3 detected a single gene or both genes belonging to same PUT assembly sequence.

n.a.: Not available.

#### TIP

The predicted polypeptides of TIP1 subfamily members ranged from 249 - 284 amino acids in length and showed 77 - 100% identity. However, 'TIP1;1, TIP1;3, and TIP1;8' and 'TIP1;5 and TIP1;14' shared 99 - 100% amino acid sequence identity (Table 1). Nine other TIP members belonged to TIP2 and TIP4 with ORF lengths ranging from 245 to 250 amino acids and 75 - 100% identity in each subgroup. Within 'TIP2;1, TIP2;2, TIP2;3 and TIP2;5', 'TIP2;6 and TIP2;7', and 'TIP4;1 and TIP4;2' we also observed very high sequence similarity (Additional file 2). The ar/R selectivity filter in TIP1 was His (H), Ile (I), Ala (A), and Val (V) with no exception; whereas the five Froger's positions were also very well conserved in all predictable sites as Thr (T), Ala (A), Ser (S), Tyr (Y), and Trp (W). Conservation in the ar/R selectivity filter and five Froger's positions provides evidence that TIP1 aquaporins likely perform similar biological functions. In other TIPs, these predicted functional sites were perfectly conserved within each subgroup and P1 - P5 were Thr (T), Ser (S), Ala (A), Tyr (Y), and Trp (W) in all TIPs (Table 2). Two conserved NPA motifs were also observed in this subfamily. Most of the full-length TIP protein sequences were predicted to be localized to the tonoplast or plasma membrane (Table 2).

#### NIP

The current study represents the first description of NIP aquaporins from allotetraploid cotton. Lengths of predicted NIP polypeptide sequences were 259 - 288 amino acids. Twelve NIP gene members were present in four subgroups with a minimum of approximately 40% identity while showing sequence identities ranging from 96 to 100% within each subgroup. Characteristic residues were less conserved in the NIP subfamily in which low sequence identity was evident across whole sequences. The NPA motifs for NIP members also showed divergence inconsistent with PIP and TIP subfamilies, with amino acid conversions from Ala (A) to Ser (S), Thr (T) or Val (V). In two NIP5 members, Ala (A) residues from both NPA motifs were converted to Ser (S) and Val (V) in the first and second NPA motifs, respectively. In the case of NIP2;1, ar/R selectivity filters were very well conserved with *Populus *NIP2. Four amino acid residues, which include Gly (G), Ser (S), Gly (G), and Arg (R), show characteristics of the NIP2;1 group that have been identified as silicon transporters [37]. Apart from cotton NIP2;1 and homologues in other plants (*Populus *and rice), AtNIP2;1 was phylogenetically not closely related to the NIP2;1 as shown in Figure 1. Based on EST sequence data and PCR sequences from cDNAs and genomic DNA, two of the NIP6 subgroup members, *NIP6;1 *and *NIP6;2 *showed very high sequence similarity to *NIP6;6 *(Table 1 and Table 3).

#### SIP

The current study represents the first description of SIP aquaporins from allotetraploid cotton. Predicted SIP polypeptides had relatively short ORFs ranging from 240 to 247 amino acids (Table 1). These proteins also shared low sequence identity with a minimum 45% and all of the identified cotton SIPs were classified phylogenetically in a single SIP1 subgroup. Sites of characteristic residues were quite divergent compared to other subfamilies, which provides evidence of different solute permeability. An Ala (A) residue present in the first NPA motif was converted to Thr (T) in four SIPs (Table 2). In addition, the Asn (N) residue present in the first NPA motif of SIP1;1 was converted to Asp (D) acid. This amino acid change (Asn (N) to other amino acids) is the only case of the conversion of Asn (N) in the NPA motif among all of the cotton aquaporins.

#### XIP

Overall, XIP aquaporins represent a recently discovered subfamily and the current study represents the first characterization of the specific XIP member from upland cotton. We identified a single, full-length cotton XIP gene coding for a 302 amino acid polypeptide. This polypeptide contains a distinct ar/R selectivity filter, Ile (I), Thr (T), Val (V), and Arg (R) (Table 2). In this gene, the first NPA motif was converted to NPV. By conducting a homology search using a D-genome *G. ramondii GrXIP *EST CO092422[3] that has 88% similarity to *GhXIP1;1 *at the deduced amino acid level, two additional cotton XIP EST sequences (*GaXIP1;1 *BG443509 and *GaXIP1;2 *BQ411475) were identified. These ESTs were derived from an A-genome species *G. arboreum *with identity of 96% and 61% to *GhXIP1;1*, respectively. The identification of an additional *G. arboreum *aquaporin provides evidence that at least one additional XIP copy may be present in the tetraploid *G. hirsutum *genome.

### Gene structure

**Figure 2:**
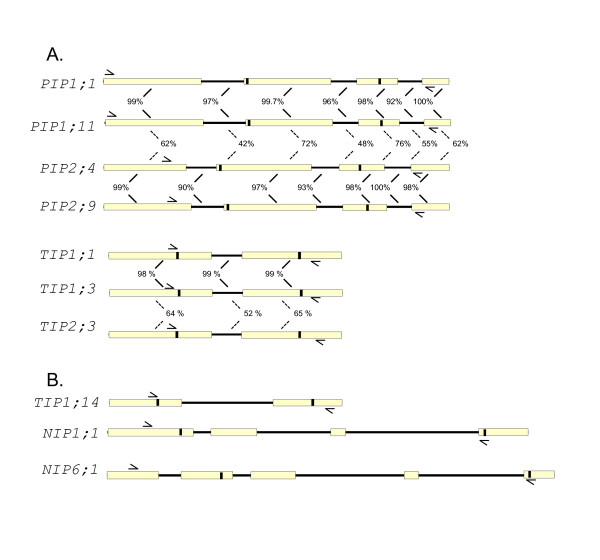
**Prediction of gene structures in PIP, TIP and NIP subgroups of cotton aquaporin genes**. Open box: exons. Black line: introns. Black shading: Two NPA motifs in loop B and loop E. A, Sequence identity of exons and introns in same position is shown as a percentage. Nucleotide similarity between same groups is indicated in solid lines and dotted lines identify similarity between different groups. B, Additional exon-intron structures are shown for *TIP1;15*, *NIP1;1 *and *NIP6;1 *that have no homologous genomic sequences available for comparison. *NIP6;1 *is partial at the C-terminal end. Positions of degenerate primer pairs used for amplifying genomic fragments are marked as half arrows.

By comparing exon-intron tandem arrays predicted from several genomic and cDNA clones of cotton aquaporin genes, several structural features of interest were observed. *PIP1;1 *and *PIP1;11 *showed very high sequence homology (99%) with identical exon-intron structures and lengths (Figure 2). This structural identity was also conserved between *PIP2;4 *and *PIP2;9*, which were 98% identical. Moreover, these two sets of pairs from different subgroups, although having lower sequence homology, appeared structurally very similar. The gene structure exhibited three introns at similar locations, and lengths of exons and introns were almost identical as reported earlier [9,16]. Consistent with the observation of Liu et. al. [20], two NPA motifs were found in the beginning of the second exon and in the middle of the third exon, respectively.

Exons were well conserved within each aquaporin subfamily (98 - 99% on average) compared to introns (94 - 95% on average). Meanwhile, in spite of highly similar exon-intron structure, the average identity between *PIP1;11 *and *PIP2;4 *was only 70% in four exons and 48% in three introns (Figure 2A). This structural and sequence similarity was also found in the TIP subfamily. Genomic sequences of two closely related genes, *TIP1;1 *and *TIP1;3*, showed these genes had at least one intron separating two exons. One TIP2 genomic clone identified in this study (*TIP2;3*) also had a similar exon-intron structure to TIP1 genes; however, their nucleotide sequences shared less than 65% identity (Figure 2A). This finding provides evidence that aquaporin genes have diverged at the nucleotide sequence level, while preserving structural characteristics such as the exon-intron splicing junction. Because of the location of primers used for genomic DNA amplification in our study, only one intron sequence was predicted in TIP1 and TIP2. Previously, Ferguson et al. [21] and Liu et al. [20] reported that *GhγTIP1 *(*GhTIP1;2*) and *Ghδ-TIP *(*GhTIP2;1*) contained two introns in their genomic sequences. Figure 2B also showed three additional genomic clones with complex gene structure. Particularly, *NIP1;1 *and *NIP 6;1 *genes had long introns with 3 and 4 exons, respectively. *NIP6;1 *and *NIP6;2 *also shared very high identity between partial intron sequences (data not shown).

### Expression analysis

Previous reports have suggested that aquaporins are present in all plant tissues and are regulated temporally and spatially depending on developmental stage and environmental conditions [38,39]. Because the level of mRNA transcript is an important factor of gene regulation, we initially examined the expression of a set of aquaporin genes by semi-quantitative RT-PCR.

**Figure 3:**
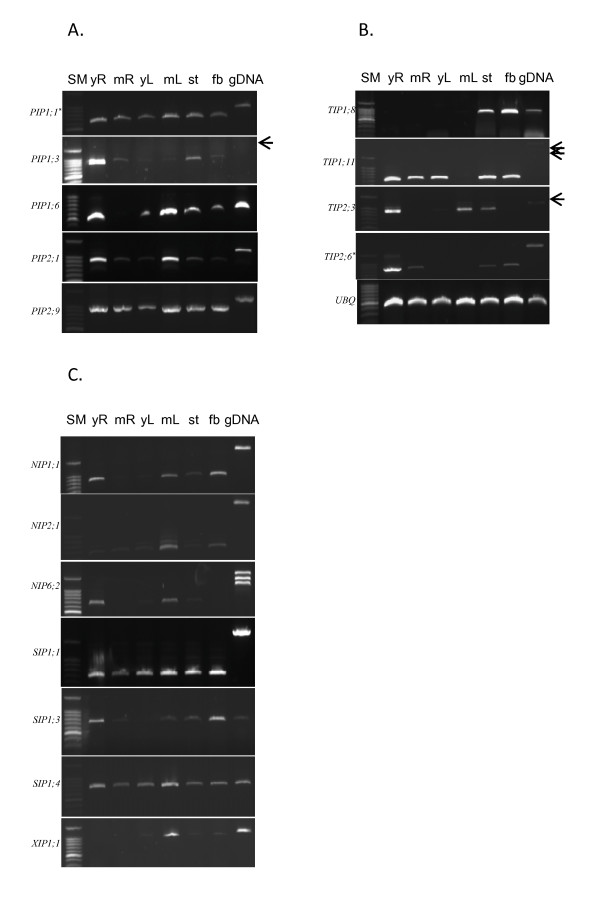
**Expression patterns of aquaporin genes among various tissues in cotton**. RT-PCR was performed to amplify aquaporin gene members in the PIP subfamily (A), TIP subfamily (B) and subfamilies of NIP, SIP, and XIP (C). The *ubiquitin *(*UBQ*) gene was used as a loading control for this experiment and some faint gDNA bands are indicated by arrows. Purity of total RNA was confirmed by a negative RT reaction (data not shown). yR: young root, mR: mature root, yL: young leaf, mL: mature leaf, st: stem, fb: 10 - 15 days post anthesis (DPA) fiber. SM denotes 100 bp DNA size marker.*: RT-PCR detects two members of aquaporin genes (*see Table *3).

Six genes from PIP (*PIP1;1*, *PIP1;14*, *PIP1;3*, *PIP1;6*, *PIP2;1*, and *PIP2;9*), 5 genes from TIP (*TIP1;8*, *TIP1;11*, *TIP2;3*, *TIP2;6*, and *TIP2;7*), 3 each from NIP (*NIP1;1*, *NIP2;1*, and *NIP6;2*) and SIP (*SIP1;1*, *SIP1;3*, and *SIP1;4*), and *XIP1;1 *were analyzed to compare the abundance of mRNA transcripts in various tissues. The plant tissues represented were harvested from two developmental stages of roots and leaves, young stems, and fibers (Figure 3). Most of the PIP members were expressed in all of the tissues tested, except *PIP1;6*, which was not expressed in the mature root. *PIP1;1*, *PIP1;14 *and *PIP2;9 *were abundant in all tissues and constitutively expressed compared to other PIP members (Figure 3A). *PIP1;6 *and *PIP2;1 *showed higher expression levels in young root and mature leaf. For the TIP aquaporins, the levels of mRNA transcripts were variable across tissues and some were expressed tissue specifically. *TIP1;8 *was detected only in stem and fiber while *TIP2;3 *and *TIP2;6 *(*TIP2;7*) were most abundant in young root (Figure 3B). In general, NIP aquaporin genes were less abundantly expressed. Among the NIPs, *NIP1;1 *had relatively higher expression in young root and fiber, *NIP2;1 *in mature leaf, and *NIP6;2 *in young root and mature leaf. *SIP1;1 *and *SIP1;4 *aquaporin genes were constitutively and highly expressed in all tissues; whereas *SIP1;3 *was abundant in young root and fiber. Interestingly, although *XIP1;1 *was highly expressed in mature leaf tissues, no transcript was detected in roots (Figure 3C).

### Analysis of cotton PUT contig sequences

Assembled EST data provides a useful resource for the identification and analyses of nucleotide sequence information. This is especially true for many polyploid crops such as cotton and wheat [40], when whole genomic sequences are not available. It is also important to use this information with care - especially when dealing with multigene families. Because sequences with high similarity are often assembled together into a single contig, it can result in inadvertent assembly-induced sequence recombination [41]. After we initially identified aquaporin candidate genes, each of those genes were blasted against PlantGDB-assembled unique transcripts (PUT) sequences in PlantGDB http://www.plantgdb.org/. PlantGDB is a database of plant EST sequences that are assembled into contigs representing tentative unique genes [42].

Initially, PUT contigs corresponding to each aquaporin gene were identified. Then, to minimize potential errors in contig assemblies, sequence alignments of each PUT contig were visually inspected. This was accomplished, as suggested by Dong et. al. [42], by examining and reconstituting all sets of EST populations. Two interesting phenomena were uncovered from this analysis as follows. First, 10 PUT sequence contigs were identified as mixtures of two or three sub-contigs that could be separated and matched to an individual aquaporin gene (Table 3). Second, in seven out of ten PUTs analyzed, a subset of ESTs was differentiated from one another according to their tissue origin. Accordingly, we separated those PUT sequences into two or three individual aquaporin genes. For example, PUT41616 consisted of 18 ESTs which were divided into two aquaporins, *PIP1;1 *and *PIP1;14*. Ten of the 16 ESTs for *PIP1;1 *originated from fiber ESTs. The remaining two ESTs belonged to *PIP1;14 *and originated from stem ESTs (Additional file 4). A similar pattern was found for *TIP2;1 *and *TIP2;3*. In fact, both genes were isolated independently by PCR cloning and were matched to different ESTs. However, by comparing PUT contig data, the EST sequences representing *TIP2;1 *and *TIP2;3 *were shown to be a part of the same PUT contig, PUT3730. As a result of visual analysis of PUT3730, it appeared that 16 ESTs represented *TIP2;1 *while *TIP2;3 *was reconstituted by 12 other ESTs isolated from stem (5 ESTs) and root (2 ESTs). RT-PCR analysis also showed that *TIP2;3 *was highly abundant in young root, stem and leaf tissues, which supported the result of *TIP2;3 *abundance from the PUT analysis. From this analysis, it was possible to annotate cotton aquaporin genes more precisely. Furthermore, comparable expression data were obtained (Table 3) that supported RT-PCR data (Figure 3).

## Discussion

### Highly similar and divergent cotton aquaporin genes

The significance of the multigene family of aquaporin transmembrane proteins is emerging from studies aimed at optimizing water and nutrient use efficiency. This large gene family has been shown to be highly diversified in plants and thus likely harbors functionally multifaceted behaviors in plants under various growth circumstances. Since the global importance of cotton as a primary natural fiber source in production agriculture is well established, our goal in this study was to identify all the members of the aquaporin family in the cotton genome. Toward this end, combined efforts were required from bioinformatic homology search and the cloning of cDNA and genomic DNA gene fragments. Bioinformatic sequence data of cotton are limited in both EST databases and an available genomic sequence; the majority of cotton EST databases consist of sequences known to be expressed in fiber tissues [41] and thus are not sufficient in expressed sequences from other plant tissues.

Our combined approach enabled us to identify a total of 71 aquaporin genes in cotton. The number of aquaporin genes described in this study almost doubles previously reported numbers within most single species and is greater than the largest number (55 members) recently identified *Populus *aquaporin genes [7]. This increase is likely the result from highly similar aquaporin members within each aquaporin subfamily, primarily in PIP (28 in cotton vs. 15 in *Populus*) and TIP subfamilies (23 in cotton vs. 17 in *Populus*) (Figure 1 and Table 1). Meanwhile, the number of NIP and SIP aquaporin genes identified here are similar to the number identified in other plant species; hence, it is possible that more genes belonging to these subfamilies are yet to be isolated in cotton. It is also plausible that the large number of aquaporin genes identified in this study is somewhat inflated due to gene duplication, which has been reported in tetraploid cotton [35]. Accordingly, determining the genome assignment (A or D) of each identified candidate aquaporin gene will be important to investigate the evolutionary history of cotton aquaporin genes during allotetraploid formation. In the case of XIP aquaporins, thus far only one member has been cloned in tetraploid cotton, while 6 have been cloned in *Populus*. From the comparison mentioned above between cotton and *Populus *aquaporins, it is plausible that genome merger/doubling during cotton domestication affected the expansion of PIP and TIP subfamilies while NIP, SIP, and XIP subfamilies were not affected or subsequently deleted after genome duplication. From the presence of three additional XIP EST sequences in A or D genome cotton species, it is also possible that tetraploid cotton may have evolved to contain more copies of aquaporin genes belonging to XIP subfamily. Also, as shown in Table 3, when we analyzed our sequence data along with PUT assembly contig sequences, a set of aquaporin gene pairs existed with several nucleotide substitutions while conserving their amino acid sequence. In these cases, most of the amino acid sites remained unchanged in spite of nucleotide substitution. Because we cloned PCR fragments from allotetraploid cotton, each cloned pair of genes with high sequence homology might represent duplicated copies present in the A and D genomes [43].

From the preliminary analysis, it appeared that several PCR clones identified as individual sequences belonged to the same PUT assembly contig. Those PCR sequences were pairs of *TIP1;1 *and *TIP 1;3 *(for PUT295101081), *TIP2;1 *and *TIP2;3 *(for PUT3730), and *NIP6;1 *and *NIP6;2 *(for PUT83990). Interestingly, we compared the partial aquaporin fragment previously isolated by Smart et al. from cotton fiber [44], and found the fragment differed from TIP2;4 and TIP2;5 by a single amino acid. Considering the polyploid nature of upland cotton, we decided to compare all candidate aquaporin genes with PUT contig sequences and subsequently inspected the assembled data visually in each PUT. This analysis allowed us to separate genes with high sequence similarity from a unique PUT transcript into individual aquaporin genes. In the case of the *PIP1;1 *contig from PlantGDB, PUT-165a-*Gossypium*_*hirsutum*-41616 consisted of 18 ESTs. Two of the 18 ESTs [GI84144149 and GI84144380] represented a different sequence which had 4 nucleotide differences. Therefore, we were able to differentiate *PIP1;1 *and *PIP1;14 *from the original contig, PUT41616 (Additional file 4). Moreover, these two ESTs [GI84144149 and GI84144380] were derived from the same tissue (stem). This finding prompted us to differentiate EST sequences from one another in a PUT contig assembly sequence. Because of the highly conserved sequence similarity, this type of combined assembling event has been previously reported when analyzing ESTs derived from allotetraploid cotton [41]. Likewise, a PUT contig (PUT-165a-*Gossypium_hirsutum*-368101081) assembled from 13 ESTs was separated into two individual aquaporin genes. One of the two genes was *PIP2;3*, originally cloned as a PCR fragment, and the other was *PIP2;13 *which differs at 9 nucleotide positions from *PIP2;3 *(Table 3). When predicted proteins were aligned together, six of nine predicted amino acid positions remained unchanged. One of the conserved positions was a P2 Froger's position known as a residue of functional importance (data not shown) [45]. It is also interesting that all of the EST sequences for *PIP2;3 *are derived from samples containing stems while *PIP2;13 *ESTs are from a mixture of tissues.

Although we detected an XIP gene in cotton, to date, XIP genes have not been identified in monocots or *Arabidopsis*. Hence, it is plausible that functional characteristics of XIP aquaporins have not been evolutionarily conserved to the extent of other aquaporin subfamilies such as PIP and TIP. Our data indicates that *GhXIP1;1 *does not accumulate in root tissue. However, it is highly expressed in mature leaf tissue, and moderately in other aerial tissues (Figure 3C). This expression data, along with the presence of a specialized ar/R filter in XIP, implies that XIP may have different substrate specificity or affect solute transport in different manners from other aquaporin subfamilies.

### Sequence-function relationship in cotton aquaporins

An obvious question drawn from the existence of at least 71 cotton aquaporin genes is why are so many aquaporins necessary? Subcellular localization of all PIP members is predicted to reside in the plasma membrane (Table 2). Therefore, an abundant number of channel proteins, PIP1 and PIP2 would be important for movement of water and other non-polar small molecules. These PIP isoforms are known to form multimeric tetramers *in vivo *and *in vitro*. For example, PIP1 isoforms in maize and rice are not functionally expressed alone in oocytes. This defect is alleviated by the co-expression of PIP2 isoforms causing the correct localization toward the plasma membrane and/or formation of a heterotetramer [13,46,47]. Thus, the multigenic nature of aquaporins in plants might facilitate their ability to regulate transport activities for water and other small molecules by redundantly modulating the abundance or multiple pairing of aquaporin water channels as demonstrated earlier [19]. In addition, it was demonstrated that functionally distinct vacuoles were labeled with different combinations of TIP antibodies in plant cells, supporting the diversification of the TIP subfamily in relation to vacuolar differentiation [48]. It is important to note that questions have been raised against the possible roles that TIPs play as different vacuole markers [49]. Recently, using confocal microscopy, distinct subcellular localization has been detected for ten *Arabidopsis *TIPs that showed cell-type or tissue-specific expression [50].

Highly conserved residues have been shown to be functionally important for substrate filtering and gating of aquaporin channel proteins [51]. Considering the NIP subfamily of aquaporins, it is noteworthy that the predicted Si transporters in the aquaporin channel protein family belong to NIP2 aquaporin genes [52]. As demonstrated in Table 2, four amino acids (G, S, G, R) in NIP2;1 are very well conserved at the ar/R selectivity filter across plants including cotton [53]. However, *Arabidopsis *NIP2;1 is an exception, as it is impermeable to Si and structurally less conserved compared to other NIP2;1 members [54]. Hence, it appears that AtNIP2;1 is phylogenetically not closely related to cotton NIP2;1, PtNIP2;1, and OsNIP2;1. Further investigations on the role of NIP2;1/silicon transporters in cotton and other dicot plants are needed since the Si transporter activity of NIP2;1 has been studied predominantly in monocots.

In addition, by comparing 153 MIPs among plants and animals, it has been demonstrated that five amino acid residues were distinguishable between aquaporin and glycerol channel proteins [55]. The importance of these residues was partially confirmed by modifying two residues in P4 and P5 sites of an insect aquaporin [56]. Therefore, the ar/R selectivity filter and Froger's position mentioned in Table 2 will provide a basis to understand a broad spectrum of aquaporin activities in cotton.

## Conclusions

In this study, we demonstrated that the cotton aquaporins consist of a large and highly similar multi-gene family phylogenetically divided into 5 subfamilies. The members of this gene family represent potential targets to modify the water use properties of cotton and may provide a target to manipulate water/nutrient uptake and photosynthesis efficiency [57,58]. Despite recent progress on the functional identification of aquaporins [59-61], the contribution of each aquaporin protein on substrate uptake and plant physiology remains to be elucidated. This will be achieved through sophisticated approaches such as global expression studies, knock-out experiments, and promoter analyses as well as substrate specificities under various physiological conditions in relation to water balance and nutrient uptake in cotton and other plant systems.

## Methods

### Plant Materials and Growth Conditions

Young leaf, stem, and root tissues were harvested from 1 month old plants (*G. hirsutum*, cv. TM-1) grown in a growth chamber (16 h light/8 h dark, 35°C/26°C). TM-1 was selected as it is considered the *G. hirsutum *genetic standard and has been maintained by repeated self-pollination since its release in 1970 [62]. Mature leaf and root tissues of TM-1 were harvested from field-grown cotton plants at the flowering stage. Fiber tissues were obtained 15-days post anthesis (DPA) from field grown plants. Leaf, stem and root tissues were frozen with liquid nitrogen and preserved at - 80°C before grinding. Fibers were submerged in RNA-*later *solution (Ambion) and stored at 4°C until RNA isolation.

### RNA and DNA Isolation

All tissues were ground extensively in liquid nitrogen using a mortar and pestle. Following the general procedure of Wan and Wilkins, RNA was isolated using the XT buffer system with the addition of chloroform/iso-amyl alcohol extractions and LiCl precipitation steps [63]. For RT-PCR, 1 μg of total RNA was treated with Turbo DNase (Ambion) followed by phenol extraction and alcohol precipitation. For 5'- and 3'-RACE (Rapid Amplification of cDNA Ends) PCR, mRNAs were purified using the OligoTex mRNA purification kit (Qiagen) from each of 20 μg of total RNAs of mixed tissues (young and mature roots and leaves, young stem). Purified mRNA was treated with Turbo DNase followed by phenol extraction and alcohol precipitation.

Genomic DNA was also isolated from mature leaf tissue following a modification of the RNA isolation procedure. Briefly, 0.6 volume of isopropyl alcohol was mixed with the supernatant obtained from the LiCl RNA precipitation step as above. After incubation at - 20°C for 1 hour, the precipitated pellet was dissolved in 0.9 ml of water and then 1/3 volume of 5 M potassium acetate solution was added followed by 30 min incubation at -20°C. After centrifugation, DNA was precipitated by adding 2/3 volume of isopropyl alcohol to the supernatant and incubating for 1 hour at - 20°C. After washing with 70% alcohol, the dried pellet was resuspended in 1 ml TE buffer and DNA concentration was measured with a UV-spectrophotometer (DU 730, Beckman Coulter).

### PCR amplification, cloning and sequencing

Using sequence alignment analysis of known aquaporin genes from cotton and several other plants, conserved sequence regions were selected for use in designing degenerate primers in the forward and reverse direction (Additional file 1). A suitable reaction condition for each primer set was determined using genomic DNA prior to the actual reaction. Amplification of aquaporin genes was performed by RT-PCR and genomic DNA PCR using a DNA thermocycler (DNA Engine Dyad, Bio-Rad). RT- PCR reactions were performed with M-MuLV Reverse Transcriptase and oligo d(T) primer. High fidelity Pfusion hot start DNA polymerase was used to amplify desired fragments with degenerate primers following the manufacturer's instruction (NEB). For each primer set, a touch-down PCR condition was employed with various annealing temperatures. Twenty ng of genomic DNA or 1/20 volume of 1st-strand cDNA product was added as a template in a 20 μl PCR reaction volume. PCR products were electrophoresed and size separated in EtBr-containing agarose gels (0.8% - 1.2%) and desired bands were recovered under UV light using an Alpha Imager 3400 (Alpha Innotech).

Gene-cleaned PCR fragments were ligated to pCR4 Blunt-TOPO vectors followed by subsequent transformation into TOP10 *E*. *coli *competent cells. After colony PCR and the selection of positive clones, plasmids were isolated from bacterial clones grown in 96-well plates and bi-directional sequencing was performed using M13 forward and M13 reverse primers. For the 5' and 3' RACE PCR, an RNA adaptor was ligated to the 5' end of purified and decapped full-length messenger RNA (200 ng). Following, the ligation product was used for first strand cDNA synthesis. After initial and nested PCR amplification reactions (GeneRacer Kit, Invitrogen), amplified RACE PCR products were cloned and sequenced as above for the identification of cDNA ends.

Three to eleven recombinant plasmids per PCR fragment were subjected to bi-directional sequencing allowing for repeated isolation of identical clones from different sources of PCR fragments corresponding to about 660 sequencing reactions (Table 1). For the expression study, RT-PCR was performed similarly as mentioned above using a set of gene-specific primers. Primers used for the expression study and RACE-PCR are available upon request.

### Homology search and sequence analysis

Sequence information from NCBI (National Center for Biotechnology Information, http://www.ncbi.nlm.nih.gov/) was used for various BLAST searches, including BLASTN, BLASTX, BLASTP, and TBLASTN. Using combinations of these blast homology searches, several known cotton aquaporin genes were queried against the cotton EST database to identify expressed cotton (*G. hursutum*) aquaporin sequences. Sequences obtained were analyzed by 6-frame translation to distinguish full-length, intact ORFs from partial or pseudogene-like incomplete sequences. To identify additional cotton aquaporin genes, several *Arabidopsis *aquaporin genes including members belonging to NIP and SIP subfamilies were also blasted by repeating homology searches. The top three to five intact EST sequences were chosen as candidate aquaporin genes from each homology comparison (E value below e^-50 ^for the BLASTN). Genomic DNA and cDNA sequences obtained from sequencing of amplified clones were subjected to BLAST analyses and only clones identified as aquaporins with intact coding sequences were considered further. Genomic DNA clones were compared with ESTs, known aquaporin genes, and cDNA clones obtained in this study to determine intron-exon linearity by positioning.

We also used PlantGDB-assembled unique transcripts (PUT) data which provided sequence contigs assembled by multiple alignment using EST sequences from NCBI. All predicted amino acid sequences from cotton aquaporin genes (ESTs, cloned cDNAs and exon contigs from cloned genomic DNA) were used for phylogenetic analysis along with other plant aquaporin genes using CLUSTAL W2 http://www.ebi.ac.uk/Tools/clustalw2/index.html and TreeView program. The reliability of branches in resulting trees was supported with 1,000 bootstrap resamplings.

## Abbreviations

PCR: polymerase chain reaction.

## Authors' contributions

W.P. participated in the experimental design, performed experiments, analyzed data, and wrote the manuscript. B.E.S. performed DNA sequencing and participated in writing the manuscript. P.J.B. participated in the experimental design and writing the manuscript. B.T.C participated in the experimental design, supervised all procedures, analyzed data and wrote the manuscript. All authors have read and contributed to the writing of the manuscript.

## Supplementary Material

Additional file 1 **Degenerate primers used in this study**. Sequences, positions, and degeneracy of primers are indicated. Click here for file

Additional file 2 **Similarity within each aquaporin subgroup in cotton**. The identity of deduced amino acid sequences was compared for all aquaporin subfamilies except XIP. Click here for file

Additional file 3 **Multiple sequence alignment of cotton aquaporins**. Deduced amino acid sequences were aligned using the CLUSTALW 2.01 program. NPA motifs, bold black (italic in NPA denotes non-conserved residues); ar/R filters (H2, H5, LE1, and LE2) are highlighted in yellow; Froger's 5 positions (P1 - P5) are also marked bold above alignment. Gene names with bold letters represent partial sequences. Click here for file

Additional file 4 **PUT-165a-Gossypium_hirsutum-41616 for PIP1;1 and PIP1;14**. These data were provided as an example of PUT assembly contig analysis (*See Table *3). Click here for file
