# Supplementary material for: Identification of the family of aquaporin genes and their expression in upland cotton (Gossypium hirsutum L.)
Source: BMC Plant Biol. 2010 Jul 13;10:142. doi: 10.1186/1471-2229-10-142 (PMC3095289; doi:10.1186/1471-2229-10-142)
Supplement: Additional file 1 — Degenerate primers used in this study. Sequences, positions, and degeneracy of primers are indicated. [file 1471-2229-10-142-S1.PDF]

Additional file 1. Degenerate primers used in this study. Sequences, positions, and degeneracy of primers are indicated.

primer	sequence	position	degeneracy
PIP F1d	ATGGAGGG(G/T/C)AA(G/A)GA(G/A)GA(G/A)GATG	Start	24
PIP F2d	AAGGACTA(T/C)(G/C)(A/T)(G/T)GA(T/C)CCTCC(G/A)C	N terminal	64
PIP F3d	AT(T/C)GC(A/T)TGGGC(T/C)TT(T/C)GGTGG	TM 2	16
PIP R1d	G(G/C)(A/T)CCAATGAA(G/A/T/C)GG(G/T)CC(C/A)ACC	TM 6	64
PIP R2d	CC(G/A/T)ACCCAGAA(G/A/T)ATCCA(G/A/T)TG(G/A/T)T	L 5/3rd splicing site	81
PIP R3d	CC(G/A)GT(G/A/T)CC(G/A)GTGAT(G/T/C)GG(G/A)AT	TM 5	72
TIP F1d	GG(T/C)TC(G/A/C)GG(A/C)ATGGC(T/C)TT(T/C)AA(T/C)A	TM1/1st splicing site	96
TIP F2d	AACAT(A/C)TC(A/C)GG(T/C)GG(T/C)CAT(G/T/C)T	TM 2	48
TIP F3d	(A/C)ACAT(A/T/C)TC(A/T/C)GG(T/C)GG(A/T/C)CA(T/C)(G/T)T	TM 2	432
TIP R1d	CCA(G/A)TGGTT(G/T)(G/T)CCCA(G/T/C)G(A/T)CCA	L 5	48
TIP R2d	GG(A/T/C)CC(A/C)(G/A)CCCA(G/A)TA(G/A)A(T/C)CCA	TM 6	96
TIP R3d	GG(A/T/C)GC(G/A/C)(G/A)(G/C)CCA(G/A)TA(G/C)A(T/C)CCA	TM 6	288
NIP F1d	CC(G/T) G(C/T)A AC(T/A) CCG GG(A/G) AC(G/T) CC(A/T) G	N terminal	64
NIP F2d	GCA GAG (G/A)(T/C)G (T/A)T(A/G) G(G/C)(C/A) AC(G/C) TAC	AEF domain	128
NIP R1d	CC (C/T)A(A/T) (A/G)CT CCT (T/C)(G/A)C (T/C)GG (A/G)TT CA	L2(NPA)	128
NIP R2d	CT (C/T)AC (T/A)GG (A/G)TT CAT (C/T)GA A(C/G)C AC	L2 (NPA)	32
SIP F1d	ATG GGA (G/A)T(T/C) AT(C/A) A(C/A)G (T/A)(T/C)(G/A) GCC AT	Start	128
SIP F2d	G A(T/A)G (G/C)G(T/A) (C/A)C(A/C) ATA A(A/C)G AT(T/A) GC	Start	128
SIP R1d	AC (A/G)TA TGC CCA (A/G/T)CC (G/A)AA (G/A/T)GC (A/C)	L5 (NPA)	72
SIP R2d	AT CCA (A/G)TA AA(C/T) (A/G)TA A(A/T)A (G/C)(T/A)G	TM 6	64
XIP F1d	GTCC(G/A)(G/A)CC(T/C)TTTG(T/C)(A/T/C)(G/T)C(T/C)ACG	N terminal	192
XIP F2d	ACT(T/C)(T/C)TGGCAGAG(G/C)T(T/C)(T/C)TTGG	N terminal	32
XIP R1d	GGCTTG(A/C)CG(T/C)(T/C)T(G/A)TCAAA(A/T)GC	Loop D	32
XIP R2d	C(T/C)CGTCTCGAG(T/C)CC(G/T)AT(A/C)(G/A)CA	TM 4	32
