# Supplementary material for: Identification of the family of aquaporin genes and their expression in upland cotton (Gossypium hirsutum L.)
Source: BMC Plant Biol. 2010 Jul 13;10:142. doi: 10.1186/1471-2229-10-142 (PMC3095289; doi:10.1186/1471-2229-10-142)
Supplement: Additional file 2 — Similarity within each aquaporin subgroup in cotton. The identity of deduced amino acid sequences was compared for all aquaporin subfamilies except XIP. [file 1471-2229-10-142-S2.PDF]

Additional file 2. Similarity within each aquaporin subgroup in cotton. The identity of deduced amino acid sequences was compared for all aquaporin subfamilies except XIP.

Cotton PIP1

	PIP1;1	PIP1;14	PIP1;11	PIP1;10†	PIP1;7	PIP1;8	PIP1;9	PIP1;6	PIP1;2	PIP1;12†	PIP1;5	PIP1;13†	PIP1;3	PIP1;15	PIP1;4
PIP1;1	**–**	**100**	**99**	96	95	92	90	87	88	88	83	89	88	88	87
PIP1;14			**99**	96	95	92	90	87	88	88	83	89	88	88	87
PIP1;11				96	94	91	89	87	88	88	84	89	88	88	88
PIP1;10†					98	92	88	85	90	90	90	90	90	90	89
PIP1;7						90	88	86	87	88	82	89	88	88	87
PIP1;8							92	85	86	86	86	86	86	86	86
PIP1;9								83	84	83	84	83	83	83	83
PIP1;6									87	86	**82**	87	87	87	86
PIP1;2										95	94	97	96	96	95
PIP1;12†											95	98	98	98	98
PIP1;5												95	91	90	90
PIP1;13†													**100**	**99**	98
PIP1;3														**99**	**99**
PIP1;15															**99**
PIP1;4															**–**

Cotton PIP2

	PIP2;1	PIP2;13	PIP2;3	PIP2;12	PIP2;2	PIP2;10	PIP2;8	PIP2;11	PIP2;4	PIP2;9	PIP2;6	PIP2;5	PIP2;7
PIP2;1	**–**	94	94	92	92	79	74	79	79	79	78	78	79
PIP2;13			**99**	92	93	80	75	79	79	80	79	79	79
PIP2;3				92	92	80	74	79	78	79	78	78	78
PIP2;12					**100**	79	74	77	77	78	76	76	77
PIP2;2						79	74	77	77	78	76	76	77
PIP2;10							**71**	76	76	76	76	77	77
PIP2;8								82	83	83	81	81	81
PIP2;11									**100**	99	94	94	93
PIP2;4										98	93	93	93
PIP2;9											94	94	94
PIP2;6												99	99
PIP2;5													**100**
PIP2;7													**–**

Cotton TIP1

	TIP1;1	TIP1;3	TIP1;8	TIP1;4	TIP1;6†	TIP1;14	TIP1;5	TIP1;11†	TIP1;10†	TIP1;13†	TIP1;12†	TIP1;7	TIP1;2	TIP1;9†
TIP1;1	**–**	**100**	**99**	92	91	91	91	87	85	85	84	79	78	84
TIP1;3			**99**	92	91	91	91	87	85	85	84	79	78	84
TIP1;8				91	90	90	90	86	85	85	84	78	**77**	83
TIP1;4					98	90	90	88	84	85	85	79	78	83
TIP1;6†						89	89	88	84	85	85	78	77	83
TIP1;14							**99**	87	85	84	85	79	78	83
TIP1;5								87	85	84	85	79	78	83
TIP1;11†									93	93	92	92	91	91
TIP1;10†										98	97	97	98	98
TIP1;13†											98	98	98	98
TIP1;12†												**99**	97	97
TIP1;7													98	97
TIP1;2														**99**
TIP1;9†														**–**

Cotton TIP2-TIP4

	TIP2;1	TIP2;3	TIP2;2†	TIP2;4†	TIP4;1	TIP4;2	TIP2;6	TIP2;7	TIP2;5†
TIP2;1	**–**	**99**	**99**	94	53	53	77	77	**99**
TIP2;3			98	94	54	54	78	78	**99**
TIP2;2†				94	53	53	77	77	**99**
TIP2;4†					59	59	76	75	94
TIP4;1						**100**	**52**	**52**	61
TIP4;2							**52**	**52**	61
TIP2;6								**99**	81
TIP2;7									81
TIP2;5†									**–**

Cotton NIP

	NIP1;1	NIP1;3†	NIP1;2†	NIP2;1	NIP5;1†	NIP5;2†	NIP6;1†	NIP6;6†	NIP6;2†	NIP6;3†	NIP6;4†	NIP6;5†
NIP1;1	–	98	98	44	**43**	45	45	44	43	36	36	39
NIP1;3†			**99**	50	50	48	55	54	55	53	53	50
NIP1;2†				50	50	48	54	54	54	53	53	50
NIP2;1					39	41	42	40	40	33	33	37
NIP5;1†						97	69	70	68	59	59	67
NIP5;2†							73	75	72	72	72	74
NIP6;1†								**100**	98	98	98	**99**
NIP6;6†									**99**	98	98	**99**
NIP6;2†										96	98	**99**
NIP6;3†											98	97
NIP6;4†												98
NIP6;5†												–

Cotton SIP

	SIP1;1	SIP1;2	SIP1;4	SIP1;7†	SIP1;3	SIP1;5†	SIP1;6†
SIP1;1	–	73	**46**	46	47	45	45
SIP1;2			48	48	52	49	48
SIP1;4				97	58	58	58
SIP1;7†					59	59	58
SIP1;3						**98**	91
SIP1;5†							92
SIP1;6†							–

†: Partial sequence.
