# Supplementary material for: Identification of the family of aquaporin genes and their expression in upland cotton (Gossypium hirsutum L.)
Source: BMC Plant Biol. 2010 Jul 13;10:142. doi: 10.1186/1471-2229-10-142 (PMC3095289; doi:10.1186/1471-2229-10-142)
Supplement: Additional file 3 — Multiple sequence alignment of cotton aquaporins. Deduced amino acid sequences were aligned using the CLUSTALW 2.01 program. NPA motifs, bold black (italic in NPA denotes non-conserved residues); ar/R filters (H2, H5, LE1, and LE2) are highlighted in yellow; Froger's 5 positions (P1 - P5) are also marked bold above alignment. Gene names with bold letters represent partial sequences. [file 1471-2229-10-142-S3.PDF]

Additional file 3. Multiple sequence alignment of cotton aquaporins. Deduced amino acid sequences were aligned using the CLUSTALW 2.01 program. NPA motifs, bold black (italic in NPA denotes for non-conserved residues); ar/R filers (H2, H5, LE1, and LE2) are highlighted in yellow; Froger's 5 positions (P1 – P5) are also marked bold above alignment. Gene names with bold letters represent partial sequences.

PIP subfamily

```
PIP1_1          MEGKEEDVRLGANKFTERQPIGTAAQSQDDGKDYTEPPPAPFFEPGELTSWSFYRAGIAE 60
PIP1_2          MEGKEEDVKLGANKFSERQPIGTSAQTD---KDYKEPPAAPFFEPGELKSWSFYRAGIAE 57
PIP1_3          MEGKEEDVKLGANKFSERQPIGTSAQTD---KDYKEPPPAPLFEPGELKSWSFYRAGIAE 57
PIP1_4          MEGKEEDVKLGANKFSERQPIGTSAQTD---KDYKEPPPAPLFEPGELKSWSFYRAGIAE 57
PIP1_5          MEGKEEDVKLGANKFSERQPIGTSAQTD---KDYKEPPAAPFFEPGELKSWSFYRAGIAE 57
PIP1_6          MEGQGEDVRLGANKYRERQPIGTAAQTQDT-KDYKEPPAAPLFEPAELSSWSFYRAGIAE 59
PIP1_7          MEGKEEDVRLGANKFSERQPIGTAAQSQDD-KDYTEPPPAPLFEPSELTSWSFYRAGIAE 59
PIP1_8          MEGREEDVRLGANRFSERQPIGTAAQSQDEGKDYTEPPPAPFFELRELCSWSFYRAGIAE 60
PIP1_9          MEDREEDVRLGANRFSERQPIGTAAQSQDEGKDYTEPPAAPFFEPGELSSWSFYRAGIAE 60
PIP1_10         ------------------------------------------------------------
PIP1_11         MEGKEEDVRLGANKFTERQPIGTAAQTQDDGKDYSEPPPAPFFEPGELTSWSFYRAGIAE 60
PIP1_12         MEGKEEDVKLGANKFSERQPIGTSAQTD---KDYKEPPPAPLFEPGELKSWSFYRAGIAE 57
PIP1_13         MEGKEEDVKLGANKFSERQPIGTSAQTD---KDYKEPPPAPLFEPGELKSWSFYRAGIAE 57
PIP1_14         MEGKEEDVRLGANKFTERQPIGTAAQSQDDGKDYTEPPPAPFFEPGELTSWSFYRAGIAE 60
PIP1_15         MEGKEEDVKLGANKFSERQPIGTSAQTD---KDYKEPPPAPLFEPGELKSWSFYRAGIAE 57
PIP2_1          ---MAKDVEV------------GGEFQA---KDYHDPPPAPLVDAQELTQWSFYRAIIAE 42
PIP2_2          ---MAKDIEV------------GGEFQA---KDYHDPPPAPLVDAQELAKWSFYRAVIAE 42
PIP2_3          ---MAKDVEV------------GGEFQA---KDYHDPPPAPLVDGEELTKWSFYRAVIAE 42
PIP2_4          ---MSKEVSE-------------EGQGR---KDYVDPPPAPLIDMAELKSWSFYRA---- 37
PIP2_5          ---MSKEVSE-------------EGMSR---KDYVDPPPAPLIDVAEIKLWSFYRALIAE 41
PIP2_6          ---MSKEVSE-------------EGVSR---KDYVDPPPAPLIDVAEIKLWSFYRALIAE 41
PIP2_7          ---MSKEVSE-------------EGMSR---KDYVDPPPAPLIDVAEIKLWSFYRALIAE 41
PIP2_8          -MENSRDGKV-------------QQLHG---KDYVDPPPARLLDMEELKTWSFYRALLAE 43
PIP2_9          ---MSKEVSE-------------EGQGR---KDYVDPPPAPLIDMAELKSWSFYRALIAE 41
PIP2_10         ---MTKDIETTA------EQGGGAEFSA---KDYQDPPPAPLIDLEELTKWSLYRAAIAE 48
PIP2_11         ---MAKDIEV------------GGEFQA---KDYHDPPPAPLVDAQELAKWSFYRAVIAE 42
PIP2_12         ---MSKEVSE-------------EGQGR---KDYVDPPPAPLIDMAELKSWSFYRALIAE 41
PIP2_13         ---MAKDVEV------------GGEFQA---KDYHDPPPAPLVDGEELTKWSFYRAVIAE 42
```

```
                                                            H2
PIP1_1          FVATFLFLYITILTVMGVVKEKT--------KCPTVGIQGIAWAFGGMIFALVYCTAGIS 112
PIP1_2          FVATFLFLYITVLTVMGVSQSKT--------KCATVGIQGIAWAFGGMIFALVYCTAGIS 109
PIP1_3          FVATFLFLYITVLTVMGVSQSKT--------KCTTVGIQGIAWAFGGMIFALVYCTAGIS 109
PIP1_4          FVATFLFLYITVLTVMGVSQSKT--------KCTTVGIQGIAWAFGGMIFALVYCTAGIS 109
PIP1_5          FVATFLFLYITVLTVMGVSQSKT--------KCATVGIQGIAWAFGGMIFALVYCTAGIS 109
PIP1_6          FVATFLFLYITVLTVMGVAKSST--------KCSTVGIQGIAWAFGGMIFALVYCTAGIS 111
PIP1_7          FVATFLFLYISVLTVMGVLKDKT--------KCTTVGIQGIAWAFGGMIFALVYCTAGIS 111
PIP1_8          FMATFLFLYITILTVMGVVKENT--------KCRTVGIQGIAWAFGGMIFALVYCTAGIS 112
PIP1_9          FMATFLFLYITILTVMGVVKEKT--------KCPTVGIQGIAWAFGGMIFALVYCTAGIS 112
PIP1_10         -----------------------------------------------MIFILVYCTAGIS 13
PIP1_11         FVATFLFLYITILTVMGVVKEKT--------KCPTVGIQGIAWAFGGMIFALVYCTAGIS 112
PIP1_12         FVATFLFLYITVLTVMGVSQSKT--------KCTTVGIQGIAWAFGGMIFALVYCTAGIS 109
PIP1_13         FVATFLFLYITVLTVMGVSQSKT--------KCTTVGIQGIAWAFGGMIFALVYCTAGIS 109
PIP1_14         FVATFLFLYITILTVMGVVKEKT--------KCPTVGIQGIAWAFGGMIFALVYCTAGIS 112
PIP1_15         FVATFLFLYITVLTVMGVSQSKT--------KCTTVGIQGIAWAFGGMIFALVYCTAGIS 109
PIP2_1          FIAALLFLYITVLTVIGYKSQVDPDKG--GQDCDGVGILGIAWAFGGMIFILVYCTAGIS 100
PIP2_2          FIATLLFLYITVLTVIGYKTQTDPAKG--GEDCGGVGILGIAWAFGGMIFILVYCTAGIS 100
PIP2_3          FIATLLFLYITVLTVIGYKSQVDPDKG--SDECGGVGILGIAWAFGGMIFILVYCTAGIS 100
PIP2_4          ----LLFLYVTVATVIGHKKQQD--------ACDGVGLLGIAWAFGGMIFILVYCTAGIS 85
PIP2_5          FIATLLFLYVTIATVIGHKKQHD--------ACDGVGLLGIAWAFGGMIFILVYCTAGIS 93
PIP2_6          FIATLLFLYVTIATVIGHKKQHD--------ACDGVGLLGIAWAFGGMIFILVYCTAGIS 93
PIP2_7          FIATLLFLYVTIATVIGHKKQHD--------ACDGVGLLGIAWAFGGMIFILVYCTAGIS 93
PIP2_8          FVATLLFLYILVATVIGHKKQTG--------LCDGVGPLGIAWAVGGMIFVLVYCTAGIS 95
PIP2_9          FIATLLFLYVTVATVIGHKKQQD--------ACDGVGLLGIAWAFGGMIFILVYCTAGIS 93
PIP2_10         FIATLLFLYVTVLTVIGYKVQTDPVKNTVDPDCGGVGILGIAWAFGGMIFILVYCTAGIS 108
PIP2_11         FIATLLFLYITVLTVIGYKTQTDLAKG--GEDCGGVGILGIAWAFGGMIFILVYCTAGIS 100
PIP2_12         FIATLLFLYVTVATVIGHKKQQD--------ACDGVGLLGIAWAFGGMIFILVYCTAGIS 93
PIP2_13         FIATLLFLYITVLTVIGYKSQVDPDKG--SDECGGVGILGIAWAFGGMIFILVYCTAGIS 100
```

```
                                                                      P1
PIP1_1          GGHINPAVTFGLFLARKLSLTRAIFYMVMQCLGAICGAGVVKGFMGKTQYGMLGGGANSV 172
PIP1_2          GGHINPAVTFGLFLARKLSLTRALFYMIMQCLGAICGAGVVKGFQGDNRYEMLGGGANVV 169
PIP1_3          GGHINPAVTFGLLLARKLSLTRAVFYMIMQCLGAICGAGVVKGFEGDSRYEMLGGGANVV 169
PIP1_4          GGHINPAVTFGLLLARKLSLTRAVFYMIMQCLGAICGAGVVKGFEGDSRYEMLGGGANVV 169
PIP1_5          GGHINPAVTFGLFLARKLSLTRALFYMIMQCLGAICGAGVVKGFQGDNRYEMLGGGANVV 169
PIP1_6          GGHINPAVTFGLFLARKLSLIRAVFYMIMQCLGAICGAAVVKSFQ-KTQYERLGGGANTV 170
PIP1_7          GGHINPAVTFGLFLGRKLSLTRAIFYMVMQCLGAICGAGVVKGFMGKTRYGALGGGANSV 171
PIP1_8          GGHINPAVTFGLFLARKLSFARAVFYMAMQCLGAICGAGVVKGFMGKTRYGLLGGGANAV 172
PIP1_9          GGHINPAVTFGLFLARKLSLTRAVFYMVMQCLGAICGAGVVKGFMGKTRYGLLGGGANAV 172
PIP1_10         GGHINPAVTFGLFLGRKLSLTRAIYYMVMQCLGAICGAGVVKGFMGKTRYGALGGGANSV 73
PIP1_11         GGHINPAVTFGLFLARKLSLTRAIFYMVMQCLGAICGAGVVKGFMGKTQYGMLGGGANSV 172
PIP1_12         GGHINPAVTFGLLLARKLSLTRAVFYMIMQCLGAICGAGVVKGFEGDSRYEMLGGGANVV 169
PIP1_13         GGHINPAVTFGLLLARKLSLTRAVFYMIMQCLGAICGAGVVKGFEGDSRYEMLGGGANVV 169
PIP1_14         GGHINPAVTFGLFLARKLSLTRAIFYMVMQCLGAICGAGVVKGFMGKTQYGMLGGGANSV 172
PIP1_15         GGHINPAVTFGLLLARKLSLTRAVFYMIMQCLGAICGAGVVKGFEGDSRYEMLGGGANVV 169
PIP2_1          GGHINPAVTFGLFLARKVSLVRAILYMAAQCLGAICGCGLVKAFQ-KSYYNKYGGGANSL 159
PIP2_2          GGHINPAVTFGLFLARKVSLVRAIFYMGAQCLGAICGCGLVKAFQ-NSYYNQYGGGANSL 159
PIP2_3          GGHINPAVTFGLFLARKVSLVRAIFYMAAQCLGAICGCGLVKAFQ-KSYYNKYGGGANSL 159
PIP2_4          GGHINPAVTFGLFLARKVSFIRAVAYMVSQCLGAICGVGLVKAFM-KHPYNSLGGGANTV 144
PIP2_5          GGHINPAVTFGLFLARKVSLIRAVAYMVAQCLGAICGVGLVKAFM-KSEYNSLGGGANTV 152
PIP2_6          GGHINPAVTFGLFLARKVSLIRAVAYMVAQCLGAICGVGLVKAFM-KSEYNSLGGGVNTV 152
PIP2_7          GGHINPAVTFGLFLARKVSLIRAVAYMVAQCLGAICGVGLVKAFM-KSEYNSLGGGANTV 152
PIP2_8          GGHINPAVTLGLFLARKVSLFRAVAYMVAQCLGAICGAGLAKSLM-KHPYNRLGGGTNLV 154
PIP2_9          GGHINPAVTFGLFLARKVSLIRAVAYMVSQCLGAICGVGLVKAFM-KHPYNSLGGGANTV 152
PIP2_10         GGHINPAVTFGLFVGRKVSLIRALLYMVAQCLGAICGCGLVKAFQ-KTYYNSYGGGANEL 167
PIP2_11         GGHINPAVTFGLFLARKVSLVRAIFYMGAQCLGAICGCGLVKAFQ-NSYYNQYGGGANSL 159
PIP2_12         GGHINPAVTFGLFLARKVSLIRAVAYMVSQCLGAICGVGLVKAFM-KHPYNSLGGGANTV 152
PIP2_13         GGHINPAVTFGLFLARKVSLVRAIFYMTAQCLGAICGCGLVKAFQ-KSYYNKYGGGANSL 159

                                                                               H5
PIP1_1          AHGYTKGDGLGAEIVGTFVLVYTVFSATDAKRSARDSHVP-ILAPLPIGFAVFLVHLATI 231
PIP1_2          NHGYTKGDGLGAEIIGTFVLVYTVFSATDAKRNARDSHVP-ILAPLPIGFAVFLVHLATI 228
PIP1_3          NHGYTKGDGLGAEIIGTFVLVYTVFSATDAKRNARDSHVP-ILAPLPIGFAVFLVHLATI 228
PIP1_4          NHGYTKGDGLGAEIVGTFILVYTVFSATDAKRNARDSHVP-ILAPLPIGFAVFFVHLATI 228
PIP1_5          NHGYTKGDGLGAEIIGTFVLVYTVFSATDAKRNARDSHVP-ILAPLPIGFAVFLVHLATI 228
PIP1_6          SSGYSKSSGLGAEIVGTFVLVYTVFSATDAKRNARDSHVP-ILAPLPIGFAVFLVHLATI 229
PIP1_7          NHGYTKGDGLGAEIVGTFVLVYTVFSATDAKRSARDSHVP-ILAPLPIGFAVFLVHLATI 230
PIP1_8          NPGYTKGAGLGAEIVGTFVLVYTVFSATDAKRSARDSHVP-ILAPLPIGFAVFLVHLATI 231
PIP1_9          NPDYTNGAALGAEIVGTFVLGYTVFSATDAKRSARDSHVP-ILAPLPIGFAVVLVHLATI 231
PIP1_10         NHGYTKGDGLGAEIVGTFVLVYTVFSATDAKRSARDSHVP-ILAPLPIGFAVFLVHLATI 132
PIP1_11         AHGYTKGDGLGAEIVGTFVLVYTVFSATDAKRSARDSHVP-ILAPLPIGFAVFLVHLATI 231
PIP1_12         NHGYTKGDGLGAEIVGTFILVYTVFSATDAKRNARDSHVP-ILAPLPIGFAVFLVHLATI 228
PIP1_13         NHGYTKGDGLGAEIIGTFVLVYTVFSATDAKRNARDSHVP-ILAPLPIGFAVFLVHLATI 228
PIP1_14         AHGYTKGDGLGAEIVGTFVLVYTVFSATDAKRSARDSHVP-ILAPLPIGFAVFLVHLATI 231
PIP1_15         NHGYTKGDGLGAEIVGTFILVYTVFSATDAKRNARDSHVP-ILAPLPIGFAVFLVHLATI 228
PIP2_1          ADGYSTGTGLAAEIIGTFVLVYTVFSATDPKRNARDSHVP-VLAPLPIGFAVFMVHLATI 218
PIP2_2          AEGYSTGTGLAAEIIGTFVLVYTVFSATDPKRNARDSHIPVVLAPLPIGFAVFMVHLATI 219
PIP2_3          ADGYSTGTGFAAEIIGTFVLVYTVFSATDPKRNARDSHIP-VLAPLPIGFAVFMVHLATI 218
PIP2_4          ASGYNKGTALGAEIIGTFVLVYTVFSATDPKRSARDSHVP-VLAPLPIGFAVFMVHLATI 203
PIP2_5          ATGYNKGTALGAEIIGTFVLVYTVFSATDPKRSARDSHVP-VLAPLPIGFAVFMVHLATI 211
PIP2_6          ATGYNKGTALGAEIIGTFVLVYTVFSATDPKRSARDSHVP-VLAPLPIGFAVFMVHLATI 211
PIP2_7          ATGYNKGTALGAEIIGTFVLVYTVFSATDPKRSARDSHVP-VLAPLPIGFAVFMVHLATI 211
PIP2_8          DSGYSKGTALGAEIIGTFVLVYTVFSATDPKRSARDSHVP-VLAPLPIGFAVFVVHLATI 213
PIP2_9          ASGYNNGTALGAEIIGTFVLVYTVFSATDPKRSARDSHVP-VLAPLPIGFAVFMVHLATI 211
PIP2_10         QDGFNKGTGLGAEIIGTFVLVYTVFSATDPKRSARDSHVP-VLAPLPIGFAVFMVHLATI 226
PIP2_11         AEGYSTGTGLAAEIIGTFVLVYTVFSATDPKRNARDSHIPVVLAPLPIGFAVFMVHLATI 219
PIP2_12         ASGYNNGTALGAEIIGTFVLVYTVFSATDPKRSARDSHVP-VLAPLPIGFAVFMVHLATI 211
PIP2_13         ADGYSTGTGLAAEIIGTFVLVYTVFSATDPKRNARDSHIP-VLAPLPIGFAVFMVHLATI 218
```

```
                   LE1  LE2P2 P3           P4P5
PIP1_1             PITGTGINPARSLGAAIIFNKDKGWDDHWIFWVGPFIGAALAALYHVVVIRAIPFKSK-- 289
PIP1_2             PITGTGINPARSLGAAIIYNKDHAWDDHWVFWVGPFIGAALAAVYHQIIIRAIPFKTRD- 287
PIP1_3             PITGTGINPARSLGAAIIYNKDHAWDDHWIFWVGPFIGAALAAIYHQIIIRAIPFKTRA- 287
PIP1_4             PITGTGINPARSLGAAIIYNKDHAWDDHWIFWVGPFIGAALAAIYHQIIIRGIPFKTRA- 287
PIP1_5             PITGTGINPARSLGAAIIYNKDHAWDDHWVFWGWTLHWTCTCCS---------LPPDNP-- 278
PIP1_6             PVTGTGINPARSLGAALIYNKDQAWDDHWIFWVGPFIGAALAALYHQIVIRAIPFKSK-- 287
PIP1_7             PITGTGINPARSLGAAIIFNKDKGWGGHWIFWVGPFIGAALAALYHVVVIRAIPFKSK-- 288
PIP1_8             PVTGTGINPARSLGAAIIFNKDKGWDDH-------------------------------- 259
PIP1_9             PVTGTGINPARSLGAAIIFHKGQGLG---------------------------------- 257
PIP1_10            PITGTGINPARSLGAAIIFNKDKGWDDHWIFWLGPFIG---------------------- 177
PIP1_11            PITGTGINPARSLGAAIIFNKDKGWDDHWIFWVGPFIGAALAALYHVVVIRAIPFKSK-- 289
PIP1_12            PITGTGINPARSLGAAIIYNKDHAWDDHWIFWVGPLHWT--------------------- 267
PIP1_13            PITGTGINPARSLGAAIIYNKDHAWDDHWIFWLGPFIG---------------------- 266
PIP1_14            PITGTGINPARSLGAAIIFNKDKGWDDHWIFWVGPFIGAALAALYHVVVIRAIPFKSK-- 289
PIP1_15            PITGTGINPARSLGAAIIYNKDHAWDDHWIFWVGPFIGAALAAIYHQIIIRAIPFKTRA- 287
PIP2_1             PITGTGINPARSFGAAVMFNQDKPWDDHWIFWVGPFIGAAIAAIYHQYILRAGAAKALGS 278
PIP2_2             PITGTGINPARSLGAAVIFNQDKIWDDHWIFWVGPFIGAAIAAIYHQFILRASGAKALGS 279
PIP2_3             PITGTGINPARSFGAAVIYNQDKPWDDHWMFWVGPFIGAAIAAIYHQFILRAAAVKALGS 278
PIP2_4             PITGTGINPARSFGAAVIYNNDKAWDEHWIFWVGPFVGALAAAIYHQYILRATAIKALGS 263
PIP2_5             PITGTGINPARSFGAAVIYNNDKAWDDQWIFWVGPMVGALAAAAYHQYILKAAAIKALGS 271
PIP2_6             PITGTGINPARSFGAAVIYNNDKAWDDQWIFWVGPMVGALAAAAYHQYILRAAAIKALGS 271
PIP2_7             PITGTGINPARSFGAAVIYNNDKAWDDQWIFWVGPMVGALAAAAYHQYIP---------- 261
PIP2_8             PITGTGINPARSLGAAVIYNNEKAWDDQWIFWVGPFIGAIAAAAYHQYILRATAIKALKS 273
PIP2_9             PITGTGINPARSFGAAVIYNNDKAWDDHWIFWVGPFVGALAAAIYHQYILRAAAIKALGS 271
PIP2_10            PITGTGINPARSFGAAVIYNQEK----------------SLAMING----YSGLDPSLGA 266
PIP2_11            PITGTGINPARSLGAAVIFNQDKIWDDHWIFWVGPFIGAAIAAIYHQFILRASGAKALGS 279
PIP2_12            PITGTGINPARSFGAAVIYNNDKAWDDHWIFWVGPFVGALAAAIYHQYILRAAAIKALGS 271
PIP2_13            PITGTGINPARSFGAAVIYNQDKPWDDHWIFWVGPFIGAAIAAIYHQFILRAAAVKALGS 278


PIP2_1             FRSSSAM 285
PIP2_2             FRSSFAM 286
PIP2_3             FRSSSAM 285
PIP2_4             FRSNPTN 270
PIP2_5             FRSNPSN 278
PIP2_6             FRSNPSN 278
PIP2_8             FRSSPTN 280

PIP2_9             FRSNPTN 278

PIP2_10            FVAAF-- 271
PIP2_11            FRSSFAM 286
PIP2_12            FRSNPHN 278
PIP2_13            FRSSSAM 285
```

TIP subfamily

```
TIP1_1          ---MPIRNIAVGRPEEATHPDALKAALAEFISTLIFVFAGSGSGMAFNKLTDNGATTPAG 57
TIP1_10         -----------------------------------------GSGMAFNKLTDDGSSTPAG 19
TIP1_11         -----------------------------------------GSGMAFNKLTDNGSSTPAG 19
TIP1_12         -----------------------------------------GSGMAFNKLTDDDSSTPAG 19
TIP1_13         -----------------------------------------GSGMAFNKLTDNGSSTPAG 19
TIP1_14         ---MPIRNIAVGRPEEATHPDALKAALAEFISTLIFVFAGSGSGMAFNKLTDGGATTPAG 57
TIP1_2          ---MPISRIAVGSPAEAGQADALKAALAEFISVLIFVFAGEGSGMAFNKLTDDGSSTPAG 57
TIP1_3          ---MPIRNIAVGRPEEATHPDALKAALAEFISTLIFVFAGSGSGMAFNKLTDNGATTPAG 57
TIP1_4          ---MPIRNIAVGRPEEATQPDALKAALAEFISTLIFVFAGSGSGMAFNKLTDNGATTPAG 60
TIP1_5          ---MPIRNIAVGRPEEATHPDALKAALAEFISTLIFVFAGSGSGMAFNKLTDGGATTPAG 57
TIP1_6          ---GTSRNIAVGRPEEATQPDALKAALAEFISTLIFVFAGSGSGMAFNKLTDNGATTPAG 57
TIP1_7          ---MPISRIAVGSPAEAGQADALKAALAEFISVLIFVFAGEGSGMAFNKLTDDDSSTPAG 57
TIP1_8          ---MPIRNIPVGRPEEATHPDALKAALAEFISTLIFVFAGSGSGMAFNKLTDNGATTPAG 57
TIP1_9          -----------------------------------------GSGMAFNKLTDDGSSTPAG 19
TIP2_1          -----MAGIAFGRFDDSFSLGTVKAYLAEFISTLVFVFAGVGSAIAYNKLTTDAALDPDG 55
TIP2_2          --------SARGRFDDSFSLGTVKAYLAEFISTLVFVFAGVGSAIAYNKLTTDAALDPDG 52
TIP2_3          -----MAGIAFGRFDDSFSLGSVKAYLAEFISTLVFVFAGVGSAIAYNKLTTDAALDPDG 55
TIP2_4          ------------------------------------------------------------
TIP2_5          ------------------------------------------------------------
TIP2_6          -----MVKIAFGSIGDSFSVGSLKAYLAEFIATLLFVFAGVGSAIAYGKLTSDAALDPPG 55
TIP2_7          -----MVKIAFGSIGDSFSVGSLKAYLAEFIATLLFVFAGVGSAIAYGKLTSDAALDPPG 55
TIP4_1          -----MPKIALGTSQEAAQPDCIKALVVEFITTFLFVFVGVGAAMAAD---ESGANALVG 52
TIP4_2          -----MPKIALGTSQEAAQPDCIKALVVEFITTFLFVFVGVGAAMAAD---ESGANALVG 52


                       H2
TIP1_1          LVAAALAHGFGLFVAVSVGANISGGHVNPAVTFGAFVGGNITLLRGILYWIAQLLGSTVA 117
TIP1_10         LVAAALAHALALFVAVSIGANISGGHVNPAVTFGAFVGGHITLVRSILYWIAQLLGSVVA 79
TIP1_11         LVAASVAHAFALFVAVSVGANISGGHVNPAVTFGAFVGGHITLFRSILYWIAQLLGSVVA 79
TIP1_12         LVAAALAHALALFVAVSVGANISGGHVNPAVTFGAFVGGHITLVRSILYWIAQLLGSVVA 79
TIP1_13         LVAAALAHALALFVAVSIGANISGGHVNPAVTFGAFVGGHITLVRSILYWIAQLLGSVVA 79
TIP1_14         LVAASLAHGFALFVAVSVGANISGGHVNPAVTFGAFVGGNITLLRGILYWIAQLLGSTVA 117
TIP1_2          LVAAALAHALALFVAVSIGANISGGHVNPAVTFGAFVGGHITLVRSILYWIAQLLGSVVA 117
TIP1_3          LVAAALAHGFGLFVAVSVGANISGGHVNPAVTFGAFVGGNITLLRGILYWIAQLLGSTVA 117
TIP1_4          LVAASIAHGLGLFVAVSVGANISGGHVNPAVTFGAFVGGNISLLRGILYWIAQLLGSTVA 120
TIP1_5          LVAASLAHGFALFVAVSVGANISGGHVNPAVTFGAFVGGNITLLRGILYWIAQLLGSTVA 117
TIP1_6          LVAASIAHGLGLFVAVSVGANISGGHVNPAVTFGAFVGGNISLLRGILYWIAQLLGSTVA 117
TIP1_7          LVAAALAHALALFVAVSVGANISGGHVNPAVTFGAFVGGHITLVRSILYWIAQLLGSVVA 117
TIP1_8          LVAAALAHGFGLFVAVSVGANISGGHVNPAVTFGAFVGGNITLLRGILYWIAQLLGSTVA 117
TIP1_9          LVAAALAHALALFVAVSIGANISGGHVNPAVTFGAFVGGHITLVRSILYWIAQLLGSVVA 79
TIP2_1          LVAIAVCHGFALFVAVAIGANISGGHVNPAVTFGLALGGQITILTGIFYWIAQLLGSIVA 115
TIP2_2          LVAIAVCHGFALFVAVAIGANISGGHVNPAVTFGLALGGQITILTGIFYWIAQLLGSIVA 112
TIP2_3          LVAIAVCHGFALFVAVAIGANISGGHVNPAVTFGLALGGQITILTGIFYWIAQLLGSIVA 115
TIP2_4          --------------------NISGGHVNPAVTFGLALGGQITILTGIFYWIAQLLGSIVA 40
TIP2_5          --------------------NISGGHVNPAVTFGLALGGQITILTGIFYWIAQLLGSIVA 40
TIP2_6          LVAIAVAHAFALFVGVAIAANISGGHLNPAVTFGLAIGGNITILTGLFYWVAQCLGSIVA 115
TIP2_7          LVAIAVAHAFALFVGVAIAANISGGHLNPAVTFGLAIGGNITILTGLFYWVAQCLGSIVA 115
TIP4_1          LFAVAVAHALVVGVMISAG-HISGGHLNPAVTLGLLFGGHITVVRAILYWIDQLLASSAA 111
TIP4_2          LFAVAVAHALVVGVMISAG-HISGGHLNPAVTLGLLFGGHITVVRAILYWIDQLLASSAA 111
```

```
                  P1
TIP1_1     CLLLKFATGDLAVPAFGLSSGVGVSNALVFEIVMTFGLVYTVYATAVDPKKGSLGTIAPL 177
TIP1_10    CLLLKFSTGGLTTSAFALSSGVGAWNAVVFEIVMTFGLVYTVYATAVDPKKGNIGIIAPI 139
TIP1_11    CLLLKFSTGGLTTSAFALSSGVGAWNAVVFEIVMTFGLVYTVYATAIDPKKGDLGVIAPL 139
TIP1_12    CLLLKFSTGGMTTSAFSLSSGVGAWNAVVFEIVMTFGLVYTVYATAVDPKKGNIGIIAPI 139
TIP1_13    CLLLKFSTGGMTTSAFSLSSGVGAWNAVVFEIVMTFGLVYTVYATAVDPKKGNIGIIAPI 139
TIP1_14    CLLLKFATSGLGVPAFGLSSGVGALNAFVFEIVMTFGLVYTVYATAVDPKRGNLGVIAPL 177
TIP1_2     CFLLKFSTGGMTTSAFSLSSGVGAWNAVVFEIVMTFGLVYTVYATAVDPKKGNIGIIAPI 177
TIP1_3     CLLLKFATGDLAVPAFGLSSGVGVSNALVFEIVMTFGLVYTVYATAVDPKKGSLGTIAPL 177
TIP1_4     CLLLKFATSDMTVPAFGLSSGVGVWNAVVFEIVMTFGLVYTVYATAIDPKKGDLGVIAPL 180
TIP1_5     CLLLKFATSGLGVPAFGLSSGVGALNAFVFEIVMTFGLVYTVYATAVDPKRGNLGVIAPL 177
TIP1_6     CLLLKFATSDMTVPAFGLSSGVGVWNAVVFEIVMTFGLVYTVYATAIDPKKGDLGVIAPL 177
TIP1_7     CLLLKFSTGGMTTSAFSLSSGVGAWNAVVFEIVMTFGLVYTVYATAVDPKKGNIGIIAPI 177
TIP1_8     CLLLKFATGDLAVPAFGLSSGVGVSNALVFEIVMTFGLVYTVYATAVDPKKGSLGTIAPL 177
TIP1_9     CFLLMFSTGGMTTSAFSLSSGVGAWNAVVFEIVMTFGLVYTVYATAVDPKKGNIGIIAPI 139
TIP2_1     CFLLKAVTGGLTVPIHGLGAGVGAIQGVVMEIIITFALVYTVYATAADPKKGSLGTIAPI 175
TIP2_2     CFLLKAVTGGLTVPIHGLGAGVGAIQGVVMEIIITFALVYTVYATAADPKKGSLGTIAPI 172
TIP2_3     CFLLKAVTGGLTVPIHGLGAGVGAIQGVVMEIIITFALVYTVYATAADPKKGSLGTIAPI 175
TIP2_4     CFLLKAVTGGLTVPIHGLGAGVGAIQGVVMEIIITFALVYTVYATAADPKKGSLGTIAPI 100
TIP2_5     CFLLKAVTGGLTVPIHGLGAGVGAIQGVVMEIIITFALVYTVYATAADPKKGSLGTIAPI 100
TIP2_6     CLLLQFVTNGLSVPTHGVASGMSAVGGVVMEIVITFALVYTVYATAADPKKGSLGIIAPI 175
TIP2_7     CLLLQFVTNGLSVPTHSVASGMNAVGGVVMEIVITFALVYTVYATAADPKKGSLGIIAPI 175
TIP4_1     CILLKYLTGGLNTPVHTLASGMGFLQGVIWEIILTFALLFTVYATIVDPKKGSIDGLGPM 171
TIP4_2     CILLKYLTGGLNTPVHTLASGMGFLQGVIWEIILTFALLFTVYATIVDPKKGSIDGLGPM 171
```

```
                     H5     LE1   LE2P2 P3        P4P5
TIP1_1     AIGFIVGANILAGGAFDGASMNPAVSFGPALVSWTWDNHWIYWVGPLIGGGLAGLIYEFI 237
TIP1_10    AIGFIVGANILAGGAFDGASMNPAVSFGPAVVSWTWANHW-------------------- 179
TIP1_11    AIGFIVGANILAGGAFDGASMNPAVSFGPALVSWTWANHW-------------------- 179
TIP1_12    AIGFIVGANILAGGAFDGASMNPAVSFGPAVVSWSWANHW-------------------- 179
TIP1_13    AIGFIVGANILAGGAFDGASMNPAVSFGPAVVSWTWANHW-------------------- 179
TIP1_14    AIGLIVGANILAGGAFDGASMNPAVSFGPALVSWSWENHWVYWAGPLIGGGLAGLVYEFI 237
TIP1_2     AIGFIVGANILAGGAFDGASMNPAVSFGPAVVSWTWDNHWVYWLGPFIGSAIAAIVYEVF 237
TIP1_3     AIGFIVGANILAGGAFDGASMNPAVSFGPALVSWTWDNHWIYWVGPLIGGGLAGLIYEFI 237
TIP1_4     AIGFIVGANILAGGAFDGASMNPAVSFGPALVSWSWDNHWIYWLGPLIGGGLAGLIYEFA 240
TIP1_5     AIGLIVGANILAGGAFDGASMNPAVSFGPALVSWSWENHWVYWAGPLIGGGLAGLVYEFI 237
TIP1_6     AIGFIVGANILAGGAFDGASMNPAVSFGPALVSWSWDNHWIYWLGPLIGGGLAGLIYEFP 237
TIP1_7     AIGFIVGANILAGGAFDGASMNPAVSFGPAVVSWTWDNHWVYWLGPFIGSAIAAIVYEVF 237
TIP1_8     AIGFIEGANILAGGAFDGASMNPAVSFGPALVSWTWDNHWIYWVGPLIGGGLAGLIYEFI 237
TIP1_9     AIGFIVGANILAGGAFDGASMNPAVSFGPAVVSWTWANHW-------------------- 179
TIP2_1     AIGFIVGANILAAGPFSGGSMNPARSFGPAVASGDFNGIWIYWVGPLIGGGLSGLIYGNV 235
TIP2_2     AIGFIVGANILAAGPFSGGSMNPARSFGPAVASGDFNGIWIYWVGPLIGGGLSGLIYGNV 232
TIP2_3     AIGFIVGANILAAGPFSGGSMNPARSFGPAVASGDFNGIWIYWVGPLIGGGLAGLIYGNV 235
TIP2_4     AIGFIVGANILAAGPFSGGSMNPARSFGPAVASGDFNGIWVYWAGP-------------- 146
TIP2_5     AIGFIVGANILAAGPFSGGSMNPARSFGPAVASGDFNGIWIYWAGP-------------- 146
TIP2_6     AIGFIVGANILAAGPFSGGSMNPARSFGPAVVSGNFADNWIYWVGPLVGGGLAGLIYGDV 235
TIP2_7     AIGFIVGANILAAGPFSGGSMNPARSFGPAVVSGNFADNWIYWVGPLVGGGLAGLIYGDV 235
TIP4_1     LTGFVVGANILAGGAFSGASMNPARSFGPALVSWNWTDHWVYWVGPLIGGGLAGYIYENF 231
TIP4_2     LTGFVVGANILAGGAFSGASMNPARSFGPALVSWNWTDHWVYWVGPLIGGGLAGYIYENF 231
```

```
TIP1_1        FIS-NTHEQLPTTDY--------------------------------- 251
TIP1_14       FIN-QTHEQLPHH----------------------------------- 249
TIP1_2        FIAPSTYEEVPSADF--------------------------------- 252
TIP1_3        FIS-NTHEQLPTTDY--------------------------------- 251
TIP1_4        FIN-QSHEQLPPTDY--------------------------------- 251
TIP1_5        FIN-QTHEQLPNY----------------------------------- 249
TIP1_6        FIN-QSHEQLPPTDYWKQFNFIMKNGFFPFPFSGGKVFLVEAFSFLGV 284
TIP1_7        FIAPSTYEELPSADF--------------------------------- 252
TIP1_8        FIS-NTHEQLPTTDY--------------------------------- 251
TIP2_1        FMNS-DHAPLSNDF---------------------------------- 248
TIP2_2        FMNS-DHAPLVNDF---------------------------------- 245
TIP2_3        FMNS-DHAPLSNDF---------------------------------- 248
TIP2_6        FIGSYSAAPASEDYA--------------------------------- 250
TIP2_7        FIGSYSAAPASEDYA--------------------------------- 250
TIP4_1        FIVR-THVLLPQNEAF-------------------------------- 246
TIP4_2        FIVR-THVLLPQNEAF-------------------------------- 246
```

NIP subfamily

```
NIP1_1      ---------------------------MAEISGCNGNHEVVLNVNGETTHPPPPPSSAPK 33
NIP1_2      ------------------------------------------------------------
NIP1_3      ------------------------------------------------------------
NIP2_1      -------------------------------------------MATTDVSPMDQNPTPPK 17
NIP5_1      --------------------------------------------MQMLSGDCPNVGSPHT 16
NIP5_2      ------------------------------------------------------------
NIP6_1      MENEDVPSAPSTPVTPGTPGAPLFGGFKGDHRGGFNKKSLLKSCKCFSVEDSMEEGRLPP 60
NIP6_2      ------------PATPGTPGAPLFGGFKGDHRNGFNKKSLLKSCKCFSVEDSMEEGRLPP 48
NIP6_3      ------------RVTPGTPGAPLFGGFKGDHRGGFNKKSLLKSCKCFSVEDSMEEGRLPP 48
NIP6_4      ------------PATPGTPGAPLFGGFKGDHRGGFNKKSLLKSCKCFSVEDSMEEGRLPP 48
NIP6_5      ------------PATPGTPGAPLFGGFKGDHRGGFNKKSLLKSCKCFSVEDSMEEGRLPP 48
NIP6_6      ------------PVTPGTPGAPLFGGFKGDHRGGFNKKSLLKSCKCFSVEDSMEEGRLPP 48

                                                               H2
NIP1_1      RKDSDLGFSVPFIQKLMAEVLGTYFLIFAGCAAVVVNVNNEKVVSLPG--ISMVWGLAVM 91
NIP1_2      -----------------AEAMGTYFLIFAGCAAVVVNVNNEKVVSLPG--ISIVWGLAVM 41
NIP1_3      -----------------AEVMGTYFLIFAGCAAVVVNVNNEKVVSLPG--ISIVWGLAVM 41
NIP2_1      QPSFQQHCPPDFPRKVFAETIATYCRVFVTCGSAAISSVDEHKISRLG--ASVAGGLIVT 75
NIP5_1      CFTDFPAPDVSLTRKLGAEFVGTFILIFAATAGPIVNQKYSGAETLLG--NATCAGLAVM 74
NIP5_2      -------------------FVGTFILIFAATAGPIVNQKYSGAETLLG--NAACAGLAVM 39
NIP6_1      VSCSLPPPPVSLTRKVGAEFIGTFILIFAGTATAIVNQKTQGSETLIG--LAASTGLAVM 118
NIP6_2      VSCSLPPPPVSLTRKVGAEFIGTFILIFAGTATAIVNQKTQGSETLIG--LAASTGLAVM 106
NIP6_3      VSCSLPPPPVSLTRKVGAEFIGTFILIFAGTATAIVNQKTQRSETLIGLALAASTGLAVM 108
NIP6_4      VSCSLPPPPVSLTRKVGAEFIGTFILIFAGTATAIVNQKTQRSETLIGLALAASTGLAVM 108
NIP6_5      VSCSLPPPPVSLTRKVGAEFIGTFILIFAGTATAIVNQKTQGSETLIG--LAASTGLAVM 106
NIP6_6      VSCSLPPPPVSLTRKVGAEFIGTFILIFAGTATAIVNQKTQGSETLIG--LAASTGLAVM 106

                                                               P1
NIP1_1      VLVYSLGHISGAHFNPAVTIAFATCKRFPLKQVPAYVLAQVIGSTLAAGTLRLLFSGPHD 151
NIP1_2      VLVYSLGHISGAHFNPAVTIAFATCKRFPLKQVPAYVLAQVIGSTLAAGTLRLLFSGPHD 101
NIP1_3      VLVYSLGHISGAHFNPAVTIAFATCKRFPLKQVPAYVLAQVIGSTLAAGTLRLLFSGPHD 101
NIP2_1      VMIYAVGHVSGAHMNPAVTLAFAAVRHFPWKQVPFYGAAQLTGAISASFTLRVLLH-PIK 134
NIP5_1      IIILSTGHISGAHLNPSLTIAFAACRHFPWTQVPAYIAAQVSASICASFALKGVFH-PFM 133
NIP5_2      IIILSTGHISGAHLNPSLTIAFAACRHFPWTQVPAYIAAQVSASMCASFALKGVFH-PFM 98
NIP6_1      IVILSTGHISGAHLNPAVTIAFAALKHFPRKHVPVYIGAQVMASLCAAFGLKGVFH-PMM 177
NIP6_2      IVILSTGHISGAHLNPAVTIAFAALKHFPRKHVPVYIGAQVMASLCAAFGLKGVFH-PMM 165
NIP6_3      IVILSTGHISGAHLNPTVTIAFAALKHFPRKH---------------------------- 140
NIP6_4      IVILSTGHISGAHLNPTVTIAFAALKHFPRKH---------------------------- 140
NIP6_5      IVILSTGHISGAHLNPAVTIAFAALKHFPRKHVPVYIGAQVMASLCAAFGLKGVFH-PMM 165
NIP6_6      IVILSTGHISGAHLNPAVTIAFAALKHFPRKHVPVYIGAQVMASLCAAFGLKGVFH-PMM 165

                                                               H5
NIP1_1      VFAGTSPQGSDLQAFGIEFIITFYLMFIISGVATDNRAIGELAGLAIGATVLINVMFAGP 211
NIP1_2      VFAGTSPQGSDLQAFGVEFIITFYLMFIISGVATDNRAIGELAGLAIGATVLINVMFAGP 161
NIP1_3      VFAGTSPQGSDLQAFGVEFIITFYLMFIISGVATDNRAIGELAGLAIGATVLINVMFAGP 161
NIP2_1      HVGTTSPSGSDLQALIMEIVVTFSMMFITSAVATDTKAIGELAGIAVGSAVCITSILAGP 194
NIP5_1      SGGVTVPSVSHGQAFALEFLITFNLLFVVTAVATDTRAVGELAGIAVGATVMLNILVAGP 193
NIP5_2      SGGVTVPSVSHGQAFALKFLITFNLLLVDTAVATDTRAVRELAGIAVGATVMLNILVAGP 158
NIP6_1      GGGVTVPSGGFGQAFALEFIISFNLMFVVTAVATDTRAVGELAGIAVGATVMLNILIAGP 237
NIP6_2      GGGVTVPSGGFGQAFALEFIISFNLMFVVTAVATDTRAVGELAGIAVGATVMLNILIAGP 225
NIP6_5      GGGVTVPSGGFGQAFALEFMISFNLMFVVTAVATDTRA---------------------- 203
NIP6_6      GGGVTVPSGGFGQAFALEFIISFNLMFVVTAVATDTRAVGELAGIVVGATVMLNILIAGP 225
```

```
               LE1   LE2P2  P3          P4P5
NIP1_1      ITGASMNPARSLGPAIVSNHYKGIWIYLMSPTLEAVSGAWVYNMVRYTDKPLREITKSAS 271
NIP1_2      ITGASMNPA--------------------------------------------------- 170
NIP1_3      ITGASMNPARSLG----------------------------------------------- 174
NIP2_1      ISGGSMNPARSIGPAIASGEYKGIWVYVVGPVTGTLMGAWSYNLIRMRDKPHHAISPHSS 254
NIP5_1      SSGGSMNPVRTLGPAVAAGNYRALWIYLIAPTLGAVIGSATYTLVKLREDEVDAPRQVRS 253
NIP5_2      SSGGSMNPVRTLGPAVAAGNYRALWIYLIAPTLGAVIGSATYTLVKLREDEVDAPRQVRS 218
NIP6_1      ITGASMNPVRTLGPAIAANNYKAIWVYFTAPILGSPCGCRTYT----------------- 280
NIP6_2      ITGASMNPVRTLGPAIAANNYKAIWVYFTAPILGALCGAGTYTAVKLPEEDGEKPSTVRS 285
NIP6_6      ITGASMNPV--------------------------------------------------- 234


NIP1_1      FPQSSRNSG 280
NIP2_1      SFKLR---- 259
NIP5_1      FRR------ 256
NIP5_2      FRR------ 221
NIP6_2      FRR------ 288
```

SIP subfamily

```
                                                                  H2
SIP1_1        MKGPIKIAIADMVISFMWVFCSSAFGLLTYLIATAAGVQTVRWASIVIITVVFFVFLSIF 60
SIP1_2        MR-PIKIAFGDMLITFMWVFCSSMFGLFTSWIATAIGVQAISWAPIVIITFIIFVFVFIF 59
SIP1_3        -MGVIKSAMADALLTSMWVFSMPFLKILTFEIAAFLGLRPFPLAAFFITTLLVSLMMFIF 59
SIP1_4        -MGIITIAIGDALLTSMWVFSMPLLKIFTSKIVGFLGAQTLPLATLMVSTVLVSILVMIF 59
SIP1_5        -MGVITTAMADALLTSMWVFSMPFLKILTFEIAAFLGLRPFPLAAFFITTLLVSLMMFIF 59
SIP1_6        -MGIITTAMADALLTSIWVFSMPFLRILTLEIAAFLGLRSFPLAAFFITTLLVSLMMFIF 59
SIP1_7        -MGVIKTAIGDALLTSMWVFSMPLLKIFTSKIVGFLGAQTLPLATLMVSTVLVSILVMIF 59

                                                                     P1
SIP1_1        NIIGGFLGGASFDPAATTAFYAAGSGAD-TLISMALRFPAQAAGAVGGALAIMEAIPEQY 119
SIP1_2        NIIGGFLGGASFNPTGTASFYAAGVGDD-SLISMGLRFPAQAAGAVGGALAITEVMPEQY 118
SIP1_3        TIIGNALGGATFNPTASVAFYAAGLRKDWSALSMAVRFPFQAVGGVVGVKTVLGVLPREY 119
SIP1_4        SLTGNVFGGASFNPAASMTFHVAGLKKNDSILSMAVRFPAQAAGGVAGVKAALGLIPAEY 119
SIP1_5        TIIGNALGGATFNPTASVAFYAAGLRKDWSALSMAVRFPFQAVGGVVGVKTVLGVLPREY 119
SIP1_6        TIVGNALGGATFNPTASVAFYAAGLKKDWSALSMAVRFPLQAAGGVVGVNAVLGVLPREY 119
SIP1_7        SFIGNVFGGASFNPAASMTFHVAGLKKNDSILSMAVRFPAQAAGGVAGVKAALGLIPAEY 119

                                                                  H5
SIP1_1        KHMIGGPSVKVDTHTGAFAEGVLTFAITFIVLLIMLRGPQSEIFKTWFLSIVIVALVTFG 179
SIP1_2        KHMIVAPSLKVDTHTGAIAEGVLTFVITLAVLFIILKGPKSEIFKTWLLAIATVAIVLSG 178
SIP1_3        KETIKGPSLKVDIQTGFLAEGLLTFGLCLALLVILIRGPNNPLLKLLLMAISTVGFVGRG 179
SIP1_4        SNTLTGPSLKVDLHTGAVAEGLFTFGRSLALLLIMFMGPKNGWVKQWLVSLATAILVLTG 179
SIP1_5        KETIKGPSLKVDIQTGFLAEGLLTFGLCLALLVILIRGPNNPLLKLLLMAISTVGFVGRG 179
SIP1_6        KETIKGPSLKVDMQTGILAEGLFTFGLCPALLVILVRGPNNPLLKLLLMAISTVGFVGRG 179
SIP1_7        SNTLTGPSLKVDLHTGAVAEGLFTFGRSLALLLIMFMGPKNGWVKQWLVSLATAILVLTG 179

                   LE1   LE2P2 P3             P4P5
SIP1_1        SAYTGPSMNPAFAFGWAYVHNQHDTWDHFYIYWICPFIGATLAGWVFRQFFSLP-PVK-V 237
SIP1_2        TAYTGPSMNPANAFGWAYVNNWHNTWDQFYVYWICPFIGAILAAWVFRLFFPPPSPVKKA 238
SIP1_3        ANYTGPSMNPANAFGWAYVNSWHNSWEHYYVYWMGPLIGATLAAWVFRFLFSPSSSIKEK 239
SIP1_4        SRFTGAPMNPASAFGWAYVNNRHNSWELYYVYWIGALFGATLGAWVFRFMVSLPLPPPTK 239
SIP1_5        ANYTGPSMNPANAFGWAYV----------------------------------------- 198
SIP1_6        ANYTGPSMNPANAFGWAYV----------------------------------------- 198
SIP1_7        SRFTGAPMNPARAFGWAYV----------------------------------------- 198

SIP1_1        KKT------ 240
SIP1_2        KKTRKPKRA 247
SIP1_3        KA------- 241
SIP1_4        QKKA----- 243
```
