# Supplementary material for: Identification of the family of aquaporin genes and their expression in upland cotton (Gossypium hirsutum L.)
Source: BMC Plant Biol. 2010 Jul 13;10:142. doi: 10.1186/1471-2229-10-142 (PMC3095289; doi:10.1186/1471-2229-10-142)
Supplement: Additional file 4 — PUT-165a-Gossypium_hirsutum-41616 for PIP1;1 and PIP1;14. These data were provided as an example of PUT assembly contig analysis (See Table 3). [file 1471-2229-10-142-S4.PDF]

Additional file 4. PUT-165a-Gossypium_hirsutum-41616 for PIP1;1/PIP1;14. These data were provided as an example of PUT assembly contig analysis (*See Table 3*).

```
gi|164355644+         ATG
gi|84166930+          ATG
gi|164338038+         ATG
gi|31407051+          ATG
gi|164306946+         ATG
gi|78339752+          ATG
gi|118132685+         ATG
                      ____________________________________________________________
consensus             TTTATTGTCCT-TTTGGAATTTTTGATACTAAAAAAAGGAAGCAACGAAGAAGGGAAATG
                                                                                M


                          .    :    .    :    .    :    .    :    .    :    .    :
gi|164355644+         GAGGGCAAAGAAGAGGATGTTAGATTGGGAGCCAACAAGTTCACAGAGAGGCAACCAATT
gi|84166930+          GAGGGCAAAGAAGAGGATGTTAGATTGGGAGCCAACAAGTTCACAGAGAGGCAACCAATT
gi|164338038+         GAGGGCAAAGAAGAGGATGTTAGATTGGGAGCCAACAAGTTCACAGAGAGGCAACCAATT
gi|84153628+                            GTTAGATTGGGAGCCAACAAGTTCACAGAGAGGCAACCAATT
gi|31407051+          TAGGGCAAAGAAGAGGATGTTAGATTGGGAGCCAACAAGTTCACAGAGAGGCAACCAATT
gi|164306946+         GAGGGCACAGAAGAGGATGTTAGATTGGGAGCCAACAAGTTCACAGAGAGGCAACCAATT
gi|78339752+          GAGGGCAAAGAAGAGGATGTTAGATTGGGAGCCAACAAGTTCACAGAGAGGCAACCAATT
gi|118132685+         GAGGGCAAAGAAGAGGATGTTAGATTGGGAGCCAACAAGTTCACAGAGAGGCAACCAATT
                      ____________________________________________________________
consensus             GAGGGCAAAGAAGAGGATGTTAGATTGGGAGCCAACAAGTTCACAGAGAGGCAACCAATT
                       E  G  K  E  E  D  V  R  L  G  A  N  K  F  T  E  R  Q  P  I


                          .    :    .    :    .    :    .    :    .    :    .    :
gi|164355644+         GGGACCGCGGCTCAGAGTCAAGATGACGGGAAAGACTACACTGAACCACCACCGGCTCCA
gi|84166930+          GGGACCGCGGCTCAGAGTCAAGATGACGGGAAAGACTACACTGAACCACCACCGGCTCCA
gi|164338038+         GGGACCGCGGCTCAGAGTCAAGATGACGGGAAAGACTACACTGAACCACCACCGGCTCCA
gi|84153628+          GGGACCGCGGCTCAGACTCAAGATGACGGGAAAGACTATTCTGAACCACCACCGGCTCCA
gi|31407051+          GGGACCGCGGCTCAGAGTCAAGATGACGGGAAAGACTACACTGAACCACCACCGGCTCCA
gi|164306946+         GGGACCGCGGCTCAGAGTCAAGATGACGGGAAAGACTACACTGAACCACCACCGGCTCCA
gi|78339752+          GGGACCGCGGCTCAGAGTCAAGATGACGGGAAAGACTACACTGAACCACCACCGGCTCCA
gi|118132685+         GGGACCGCGGCTCAGAGTCAAGATGACGGGAAAGACTACACTGAACCACCACCGGCTCCA
gi|109825953+                                                     CACCACCGGCTCCA
                      ____________________________________________________________
consensus             GGGACCGCGGCTCAGAGTCAAGATGACGGGAAAGACTACACTGAACCACCACCGGCTCCA
                       G  T  A  A  Q  S  Q  D  D  G  K  D  Y  T  E  P  P  P  A  P


                          .    :    .    :    .    :    .    :    .    :    .    :
gi|164355644+         TTTTTCGAGCCTGGCGAGTTAACCTCATGGTCTTTTTATAGGGCTGGGATTGCCGAGTTT
gi|84166930+          TTTTTCGAGCCTGGCGAGTTAACCTCATGGTCCTTTTATAGGGCTGGGATTGCCGAGTTT
gi|164338038+         TTTTTCGAGCCTGGCGAGTTAACCTCATGGTCTTTTTATAGGGCTGGGATTGCCGAGTTT
gi|84154773+                                                               GCCGAGTTT
gi|84153628+          TTTTTCGAGCCTGGCGAGTTAACCTCATGGTCCTTTTATAGGGCTGGGATTGCCGAGTTT
gi|31407051+          TTTTTCGAGCCTGGCGAGTTAACCTCATGGTCTTTTTATAGGGCTGGGATTGCCGAGTTT
gi|164306946+         TTTTTCGAGCCTGGCGAGTTAACCTCATGGTCTTTTTATAGGGCTGGGATTGCCGAGTTT
gi|78339752+          TTTTTCGAGCCTGGCGAGTTAACCTCATGGTCTTTTTATAGGGCTGGGATTGCCGAGTTT
gi|118132685+         TTTTTCGAGCCTGGCGAGTTAACCTCATGGTCTTTTTATAGGGCTGGGATTGCCGAGTTT
gi|109825953+         TTTTTCGAGCCTGGCGAGTTAACCTCATGGTCTTTTTATAGGGCTGGGATTGCCGAGTTT
                      ____________________________________________________________
consensus             TTTTTCGAGCCTGGCGAGTTAACCTCATGGTCTTTTTATAGGGCTGGGATTGCCGAGTTT
                       F  F  E  P  G  E  L  T  S  W  S  F  Y  R  A  G  I  A  E  F
```

```
                          .    :    .    :    .    :    .    :    .    :    .    :
gi|164355644+         GTGGCGACTTTCCTTTTCTTGTATATCACTATTTTGACTGTTATGGGAGTTGTTAAGGAA
gi|84166930+          GTGGCGACTTTCCTTTTCTTGTATATCACTATTTTGACTGTTATGGGAGTTGTTAAGGAA
gi|164338038+         GTGGCGACTTTCCTTTTCTTGTATATCACTATTTTGACTGTTATGGGAGTTGTTAAGGAA
gi|84154773+          GTGGCGACTTTCCTTTTCTTGTATATCACTATTTTGACTGTTATGGGAGTTGTTAAGGAA
gi|84153628+          GTGGCGACTTTCCTTTTCTTGTATATCACTATTTTGACTGTTATGGGAGTTGTTAAGGAA
gi|31407051+          GTGGCGACTTTCCTTTTCTTGTATATCACTATTTTGACTGTTATGGGAGTTGTTAAGGAA
gi|164306946+         GTGGCGACTTTCCTTTTCTTGTATATCACTATTTTGACTGTTATGGGAGTTGTTAAGGAA
gi|78339752+          GTGGCGACTTTCCTTTTCTTGTATATCACTATTTTGACTGTTATGGGAGTTGTTAAGGAA
gi|118132685+         GTGGCGACTTTCCTTTTCTTGTATATCACTATTTTGACTGTTATGGGAGTTGTTAAGGAA
gi|109825953+         GTGGCGACTTTCCTTTTCTTGTATATCACTATTTTGACTGTTATGGGAGTTGTTAAGGAA
                      ____________________________________________________________
consensus             GTGGCGACTTTCCTTTTCTTGTATATCACTATTTTGACTGTTATGGGAGTTGTTAAGGAA
                       V  A  T  F  L  F  L  Y  I  T  I  L  T  V  M  G  V  V  K  E


                          .    :    .    :    .    :    .    :    .    :    .    :
gi|164355644+         AAAACTAAGTGCCCAACTGT-TGGGATTCAAGGAATTGCTTGGGCTTTTGGTGGTATGAT
gi|84166930+          AAAACTAAGTGCCCAACTGT-TGGGATTCAAGGAATTGCTTGGGCTTTTGGTGGTATGAT
gi|164338038+         AAAACTAAGTGCCCAACTGT-TGGGATTCAAGGAATTGCTTGGGCTTTTGGTGGTATGAT
gi|84154773+          AAAACTAAGTGCCCAACTGT-TGGGATTCAAGGAATTGCTTGGGCTTTTGGTGGTATGAT
gi|84153628+          AAAACTAAGTGCCCAACTGT-TGGGATTCAAGGAATTGCTTGGGCTTTTGGTGGTATGAT
gi|31407051+          AAAACTAAGTGCCCAACTGT-TGGGATTCAAGGAATTGCTTGGGCTTTTGGTGGTATGAT
gi|164306946+         AAAACTAAGTGCCCAACTGT-TGGGATTCAAGGAATTGCTTGGGCTTTTGGTGGTATGAT
gi|78339752+          AAAACTAAGTGCCCAACTGT-TGGGATTCAAGGAATTGCTTGGGCTTTTGGTGGTATGAT
gi|118132685+         AAAACTAAGTGCCCAACTGT-TGGGATTCAAGGAATTGCTTGGGCTTTTGGTGGTATGAT
gi|84144380+                           TGT-TGGGATTCAAGGAATTGCTTGGGCTTTTGGTGGTATGAT
gi|164293662+                   GCCCAACTGTGTGGGATTCAAGGAATTGCTTGGGCTTTTGGTGGTATGAT
gi|109825953+         AAAACTAAGTGCCCAACTGT-TGGGATTCAAGGAATTGCTTGGGCTTTTGGTGGTATGAT
                      ____________________________________________________________
consensus             AAAACTAAGTGCCCAACTGT-TGGGATTCAAGGAATTGCTTGGGCTTTTGGTGGTATGAT
                       K  T  K  C  P  T  V   G  I  Q  G  I  A  W  A  F  G  G  M  I


                          .    :    .    :    .    :    .    :    .    :    .    :
gi|164355644+         CTTTGCTCTTGTTTACTGCACTGCTGGCATTTCAGGTGGCCATATCAACCCGGCAGTGAC
gi|84166930+          CTTTGCTCTTGTATACTGCACTGCTGGCATTTCAGGTGGCCATATCAACCCGGCAGTGAC
gi|164338038+         CTTTGCTCTTGTTTACTGCACTGCTGGCATTTCAGGTGGCCATATCAACCCGGCAGTGAC
gi|84154773+          CTTTGCTCTTGTTTACTGCACTGCTGGCATTTCAGGTGGCCATATCAACCCGGCAGTGAC
gi|84153628+          CTTTGCTCTTGTTTACTGCACTGCTGGCATTTCAGGTGGCCATATCAACCCGGCAGTGAC
gi|31407051+          CTTTGCTCTTGTTTACTGCACTGCTGGCATTTCAGGTGGCCATATCAACCCGGCAGTGAC
gi|164306946+         CTTTGCTCTTGTTTACTGCACTGCTGGCATTTCAGGTGGCCATATCAACCCGGCAGTGAC
gi|78339752+          CTTTGCTCTTGTTTACTGCACTGCTGGCATTTCAGGTGGCCATATCAACCCGGCAGTGAC
gi|118132685+         CTTTGCTCTTGTTTACTGCACTGCTGGCATTTCAGGTGGCCATATCAACCCGGCAGTGAC
gi|164345171+                                  GGCATTTCAGGTGGCCATATCAACCCGGCAGTGAC
gi|84144380+          CTTTGCTCTTGTTTACTGCACTGCTGGCATTTCAGGTGGCCATATCAACCCGGCAGTGAC
gi|164255409+         CTTTGCTCTTGTCTACTGCACTGCTGGCATTTCAGGTGGCCATATCAACCCGGCAGTGAC
gi|164293662+         CTTTGCTCTTGTTTACTGCACTGCTGGCATTTCAGGTGGCCATATCAACCCGGCAGTGAC
gi|109825953+         CTTTGCTCTTGTTTACTGCACTGCTGGCATTTCAGGTGGCCATATCAACCCGGCAGTGAC
                      ____________________________________________________________
consensus             CTTTGCTCTTGTTTACTGCACTGCTGGCATTTCAGGTGGCCATATCAACCCGGCAGTGAC
                        F  A  L  V  Y  C  T  A  G  I  S  G  G  H  I  N  P  A  V  T
```

```
                        .    :    .    :    .    :    .    :    .    :    .    :
gi|164355644+       TTTTGGACTGTTCTTGGCGAGGAAGCTGTCGTTGACGAGGGCAATTTTCTACATGGTGAT
gi|84166930+        TTTTGGACTGTTCTTGGCGAGGAAGCTGTCGTTGACGAGGGCAATTTTCTACATGGTGA
gi|164338038+       TTTTGGACTGTTCTTGGCGAGGAAGCTGTCGTTGACGAGGGCAATTTTCTACATGGTGAT
gi|84154773+        TTTTGGACTGTTCTTGGCGAGGAAGCTGTCGTTGACGAGGGCAATTTTCTACATGGTGAT
gi|84153628+        TTTTGGACTGTTCTTGGCGAGGAAGCTGTCGTTGACGAGGGCAATTTTCTACATGGTGAT
gi|31407051+        TTTTGGACTGTTCTTGGCGAGGAAGCTGTCGTTGACGAGGGCAATTTTCTACATGGTGAT
gi|164306946+       TTTTGGACTGTTCTTGGCGAGGAAGCTGTCGTTGACGAGGGCAATTTTCTACATGGTGAT
gi|78339752+        TTTTGGACTGTTCTTGGCGAGGAAGCTGTCGTTGACGAGGGCAATTTTCTACATGGTGAT
gi|118132685+       TTTTGGACTGTTCTTGGCGAGGAAGCTGTCGTTGACGAGGGCAATTTTCTACATGGTGAT
gi|164345171+       TTTTGGACTGTTCTTGGCGAGGAAGCTGTCGTTGACGAGGGCAATTTTCTACATGGTGAT
gi|84144380+        TTTTGGACTGTTCTTGGCGAGGAAGCTGTCGTTGACGAGGGCAATTTTCTACATGGTGAT
gi|164255409+       TTTTGGACTGTTCTTGGCGAGGAAGCTGTCGTTGACGAGGGCAATTTTCTACATGGTGAT
gi|164293662+       TTTTGGACTGTTCTTGGCGAGGAAGCTGTCGTTGACGAGGGCAATTTTCTACATGGTGAT
gi|109825953+       TTTTGGACTGTTCTTGGCGAGGAAGCTGTCGTTGACGAGGGCAATTTTCTACATGGTGAT
                    ____________________________________________________________
consensus           TTTTGGACTGTTCTTGGCGAGGAAGCTGTCGTTGACGAGGGCAATTTTCTACATGGTGAT
                      F  G  L  F  L  A  R  K  L  S  L  T  R  A  I  F  Y  M  V  M


                        .    :    .    :    .    :    .    :    .    :    .    :
gi|164355644+       GCAGTGTTTGGGCGCCATATGCCGGGCGGGTGTGGTGAAAGGGTTCATGGGGAAAGACTC
gi|164338038+       GCAGTGTTTGGGCGCCATATGCGGGGCGGGTGTGGTGAAAGGTTTCATGGGGAA-GACTC
gi|84154773+        GCAGTGTTTGGGCGCCATATGCGGGGCGGGTGTGGTGAAAGGTTTCATGGGGAA-GACTC
gi|84153628+        GCAGTGTTTGGGCGCCATATGCGGGGCGGGTGTGGTGAAAGGTTTCATGGGGAA-GACTC
gi|31407051+        GCAGTGTTTGGGCGCCATATGCGGGGCGGGTGTGGTGAAAGGTTTCATGGGGAA-GACTC
gi|164306946+       GCAGTGTATGGGCGCCATATGCGGGGCGGGTGTGGTGAAAGG-TTCATGGGGAA-GACTC
gi|78339752+        GCAGTGTTTGGGCGCCATATGCGGGGCGGGTGTGGTGAAAGGTTTCATGGGGAA-GACTC
gi|118132685+       GCAGTGTTTGGGCGCCATATGCGGGGCGGGTGTGGTGAAAGGTTTCATGGGGAA-GACTC
gi|82755095+                            GCGGGGCGGGTGTGGTGAAAGGTTTCATGGGGAA-GACTC
gi|84144149+                                                             GGGAA-GACTC
gi|164345171+       GCAGTGTTTGGGCGCCATATGCGGGGCGGGTGTGGTGAAAGGTTTCATGGGGAA-GACTC
gi|84144380+        GCAGTGTTTGGGCGCCATATGCGGGGCGGGTGTGGTGAAAGGTTTCATGGGGAA-GACTC
gi|164255409+       GCAGTGTTTGGGCGCCATATGCGGGGCGGGTGTGGTGAAAGGTTTCATGGGGAA-GACTC
gi|164293662+       GCAGTGTTTGGGCGCCATATGCGGGGCGGGTGTGGTGAAAGGTTTCATGGGGAA-GACTC
gi|109825953+       GCAGTGTTTGGGCGCCATATGCGGGGCGGGTGTGGTGAAAGGTTTCATGGGGAA-GACTC
                    ____________________________________________________________
consensus           GCAGTGTTTGGGCGCCATATGCGGGGCGGGTGTGGTGAAAGGTTTCATGGGGAA-GACTC
                      Q  C  L  G  A  I  C  G  A  G  V  V  K  G  F  M  G  K   T


                        .    :    .    :    .    :    .    :    .    :    .    :
gi|164355644+       AGTACGGTATGTTGGGGCGGCGGAGCTAA-CTCGGNGGCTCATGGCTACCCAAAA-AGNG
gi|164338038+       AGTACGGTATGTTGGG-CGGCGGAGCTAA-CTCGGTGGCTCATGGCTACACAAAA-GGTG
gi|84154773+        AGTACGGTATGTTGGG-CGGCGGAGCTAAACTCGGTGGCTCATGGCTACACAAAA-GGTG
gi|84153628+        AGTACGGTATGTNNGG-CGGCGGAGCTAA-CTCGGTGGCTCATGGCTACACAAAA-GGTG
gi|31407051+        AGTACGGTATGTTGGG-CGGCGGAACTAA-CTCGGTGGCTCATGGCTACACAAAAAGGTG
gi|164306946+       A-TACGGTATGTAGGG-CGGCGGAGCTAA-CTCGGTGGCTCATGGCTACACAAAA-AGTG
gi|78339752+        AGTACGGTATGTTGGG-CGGCGGAGCTAA-CTCGGTGGCTCATGGCTACACAAAA-GGTG
gi|118132685+       AGTACGGTATGTTGGG-CGGCGGAGCTAA-CTCGGTGGCTCATGGCTACACAAAA-GGTG
gi|82755095+        AGTACGGTATGTTGGG-CGGCGGAGCTAA-CTCGGTGGCTCATGGCTACACAAAA-GGTG
gi|84144149+        AGTACGGTATGTTGGG-CGGCGGAGCTAA-CTCGGTGGCTCATGGCTACACAAAA-GGTG
gi|164345171+       AGTACGGTATGTTGGG-CGGCGGAGCTAA-CTCGGTGGCTCATGGCTACACAAAA-GGTG
gi|84144380+        AGTACGGTATGTTGGG-CGGCGGAGCTAA-CTCGGTGGCTCATGGCTACACAAAA-GGTG
gi|164255409+       AGTACGGTATGTTGGG-CGGCGGAGCTAA-CTCGGTGGCTCATGGCTACACCAAA-GGTG
gi|164293662+       AGTACGGTATGTTGGG-CGGCGGAGCTAA-CTCGGTGGCTCATGGCTACACAAAA-GGTG
gi|109825953+       AGTACGGTATGTTGGG-CGGCGGAGCTAA-CTCGGTGGCTCATGGCTACACAAAA-GGTG
gi|84174539-             GGTATGTTGGG-CGGCGGAGCTAA-CTCGGTGGCTCATGGCTACACAAAA-GGTG
gi|109842739+                TGTTGGG-CGGCGGAGCTAA-CTCGGTGGCTCATGGATACGCGTA--GGTG
                    ____________________________________________________________
consensus           AGTACGGTATGTTGGG-CGGCGGAGCTAA-CTCGGTGGCTCATGGCTACACAAAA-GGTG
                    Q  Y  G  M  L  G   G  G  A  N   S  V  A  H  G  Y  T  K   G
```

```
                  .    :    .    :    .    :    .    :    .    :    .    :
gi|164355644+   -ATGGCCTTGGNG-CTGAAATTGTCGGC
gi|164338038+   -ATGGCCTTGGTG-CTGAAATTGTCGGCACCTTTGTTCTTGTTTACACTGTCTTCTCAGC
gi|84154773+    -ATGGCCTTGGTG-CTGAAATTGTCGGCACCTTTGTTCTTGTTTACACTGTCTTCTCAGC
gi|84153628+    -ATGGCCTTGGTG-CTGAAAT
gi|31407051+    GATGGCCTTGGTGGCTGAAATTG
gi|164306946+   A
gi|78339752+    -ATGGCCTTGGTG-CTGAAATTGTCGGCACCTTTGTTCTTGTTTACACTGTCTTCTCAGC
gi|118132685+   -ATGGCCTTGGTG-CTGAAATTGTCGGCACCTTTGTTCTTGTTTACACTGTCTTCTCAGC
gi|82755095+    -ATGGCCTTGGTG-CTGAAATTGTCGGCACCTTTGTTCTTGTTTACACTGTCTTCTCAGC
gi|84144149+    -ATGGCCTTGGTG-CTGAAATTGTCGGCACCTTTGTTCTTGTTTACACTGTCTTCTCAGC
gi|164345171+   -ATGGCCTTGGTG-CTGAAATTGTCGGCACCTTTGTTCTTGTTTACACTGTCTTCTCAGC
gi|84144380+    -ATGGCCTTGGTG-CTGAAATTGTCGGCACCTTTGTTCTTGTTTACACTGTCTTCTCAGC
gi|164255409+   -ATGGCCTTGGTG-CTGAAATTGTCGGCACCTTCGTTCTTGTTTACACTGTCTTCTCAGC

gi|164293662+   -ATGGCCTTGGTG-CTGAAATTGTCGGCACCTTTGTTCTTGTTTACACTGTCTTCTCAGC
gi|109825953+   -ATGGCCTTGGTG-CTGAAATTGTCGGCACCTTTGTTCTTGTTTACACTGTCTTCTCAGC
gi|84174539-    -ATGGCCTTGGTG-CTGAAATTGTCGGCACCTTTGTTCTTGTTTACACTGTCTTCTCAGC
gi|109842739+   -ATGGCCTTGGTG-CTGAAATTGTCGGCACCTTTGTTCTTGTTTACACTGTCTTCTCAGC
                ____________________________________________________________
consensus       -ATGGCCTTGGTG-CTGAAATTGTCGGCACCTTTGTTCTTGTTTACACTGTCTTCTCAGC
                  D  G  L  G   A  E  I  V  G  T  F  V  L  V  Y  T  V  F  S  A


                  .    :    .    :    .    :    .    :    .    :    .    :
gi|164338038+   CACTGACGCCAAGCGCAGCGCCCGAGACTCTCATGTCCCTATTCTGGCACCATTGCCAAT
gi|84154773+    CACTGACGCCAAGCGCAGCGCC-GTGACTCTCATGTCCCTATNCTGGCACCATTGCC
gi|78339752+    CACTGACGCCAAGCGCAGCGCCCGAGACTCTCATGTCCCTATTCTGGCACCATTGCCAAT
gi|118132685+   CACTGACGCCAAGCGCAGCGCCCGAGACTCTCATGTCCCTATTCTGGCACCATTGCCAAT
gi|82755095+    CACTGACGCCAAGCGCAGCGCCCGAGACTCTCATGTCCCTATTCTGGCACCATTGCCAAT
gi|84144149+    CACTGACGCCAAGCGCAGCGCCCGTGACTCTCATGTCCCTATTCTGGCACCATTGCCAAT
gi|164345171+   CACTGACGCCAAGCGCAGCGCCCGAGACTCTCATGTCCCTATTCTGGCACCATTGCCAAT
gi|84144380+    CACTGACGCCAAGCGCAGCGCCCGTGACTCTCATGTCCCTATTCTGGCACCATTGCCAAT
gi|164255409+   CACTGACGCCAAGCGCAGCGCCCGAGACTCTCATGTCCCTATTCTGGCACCATTGCCAAT
gi|164293662+   CACTGACGCCAAGCGCAGCGCCCGAGACTCTCATGTCCCTATTCTGGCACCATTGCCAAT
gi|109825953+   CACTGACGCCAAGCGCAGCGCCCGAGACTCTCATGTCCCTATTCTGGCACCATTGCCAAT
gi|84174539-    CACTGACGCCAAGCGCAGCGCCCGAGACTCTCATGTCCCTATTCTGGCACCATTGCCAAT
gi|109842739+   CACTGACGCCAAGCGCAGCGCCCGAGACTCTCATGTCCCTATTCTGGCACCATTGCCAAT
                ____________________________________________________________
consensus       CACTGACGCCAAGCGCAGCGCCCGAGACTCTCATGTCCCTATTCTGGCACCATTGCCAAT
                  T  D  A  K  R  S  A  R  D  S  H  V  P  I  L  A  P  L  P  I


                  .    :    .    :    .    :    .    :    .    :    .    :
gi|164338038+   TGGGTTCGCAGTGTTCTTGGTACACTTAGCCACCATCCCAATCACTGGAACTGGTATTAA
gi|78339752+    TGGGTTCGCAGTGTTCTTGGTACACTTAGCCACCATCCCAATCACTGGAACTGGTATTAA
gi|118132685+   TGGGTTCGCAGTGTTCTTGGTACACTTAGCCACCATCCCAATCACTGGAACTGGTATTAA
gi|82755095+    TGGGTTCGCAGTGTTCTTGGTACACTTAGCCACCATCCCAATCACTGGAACTGGTATTAA
gi|84144149+    TGGGTTCGCAGTGTTCTTGGTACACTTAGCAACAATCCCAATCACTGGAACTGGCATTAA
gi|164345171+   TGGGTTCGCAGTGTTCTTGGTACACTTAGCCACCATCCCAATCACTGGAACTGGTATTAA
gi|84144380+    TGGGTTCGCAGTGTTCTTGGTACACTTAGCAACAATCCCAATCACTGGAACTGGCATTAA
gi|164255409+   TGGGTTCCCAGTGTTCTTGGTACACTTAGCCACCATCCCAATCACTGGAACTGGTATTAA
gi|164293662+   TGGGTTCGCAGTGTTCTTGGTACACTTAGCCACCATCCCAATCACTGGAACTGGTATTAA
gi|109825953+   TGGGTTCGCAGTGTTCTTGGTACACTTAGCCACCATCCCAATCACTGGAACTGGTATTAA
gi|84174539-    TGGGTTCGCAGTGTTCTTGGTACACTTAGCCACCATCCCAATCACTGGAACTGGTATTAA
gi|109842739+   TGGGTTCGCAGTGTTCTTGGTACACTTAGCCACCATCCCAATCACTGGAACTGGTATTAA
                ____________________________________________________________
consensus       TGGGTTCGCAGTGTTCTTGGTACACTTAGCCACCATCCCAATCACTGGAACTGGTATTAA
                  G  F  A  V  F  L  V  H  L  A  T  I  P  I  T  G  T  G  I  N
```

```
                          .   :   .   :   .   :   .   :   .   :   .   :
gi|164338038+        CCCGGCTCGTAGTCTTGGTGCAGCCATCATCTTCAAAAGGGAAAGGGGTTGGGA-TGACA
gi|78339752+         CCCGGCTCGTAGTCTTGGTGCAGCCATCATCTTCAACAAGGACAAGGGCTGGGA-TGACC
gi|118132685+        CCCGGCTCGTAGTCTTGGTGCAGCCATCATCTTCAACAAGGACAAGGGCTGGGA-TGACC
gi|82755095+         CCCGGCTCGTAGTCTTGGTGCAGCCATCATCTTCAACAAGGACAAGGGCTGGGA-TGACC
gi|84144149+         CCCGGCTCGTAGTCTTGGTGCAGCCATCATCTTCAACAAGGACAAGGGCTGGGA-TGACC
gi|164345171+        CCCGGCTCGTAGTCTTGGTGCAGCCATCATCTTCAACAAGGACAAGGGCTGGGA-TGACC
gi|84144380+         CCCGGCTCGTAGTCTTGGTGCAGCCATCATCTTCAACAAGGACAAGGGCTGGGAATGACC
gi|164255409+        CCCGGCTCGTAGTCTTGGTGCAGCCATCATCTTCAACAAGGACAAGGGCTGGGA-TGACC
gi|164293662+        CCCGGCTCGTAGTCTTGGTGCAGCCATCATCTTCAACAAGGACAAGGGCTGGGA-TGACC
gi|109825953+        CCCGGCTCGTAGTCTTGGTGCAGCCATCATCTTCAACAAGGACAAGGGCTGGGA-TGACC
gi|84174539-         CCCGGCTCGTAGTCTTGGTGCAGCCATCATCTTCAACAAGGACAAGGGCTGGGA-TGACC
gi|109842739+        CCCGGCTCGTAGTCTTGGTGCAGCCATCATCTTCAACAAGGACAAGGGCTGGGA-TGACC
                     ____________________________________________________________
consensus            CCCGGCTCGTAGTCTTGGTGCAGCCATCATCTTCAACAAGGACAAGGGCTGGGA-TGACC
                       P  A  R  S  L  G  A  A  I  I  F  N  K  D  K  G  W  D   D

                          .   :   .   :   .   :   .   :   .   :   .   :
gi|164338038+        TTTGGGATTTCCGGGGTGGGAACAATCCTTGGTTGCAGCACTAACCAGCACTCTTAC-AC
gi|78339752+         ATTGG-ATTTTCTGGGTGGGACCATTCATTGGT-GCAGCACTAGC-AGCACTCTACC-AC
gi|118132685+        ATTGG-ATTTTCTGGGTGGGACCATTCATTGGT-GCAGCACTAGC-AGCACTCTACC-AC
gi|82755095+         ATTGG-ATTTTCTGGGTGGGACCATTCATTGGT-GCAGCACTAGC-AGCACTCTACC-AC
gi|84144149+         ATTGG-ATTTTCTGGGTGGGACCATTCATTGGT-GCAGCACTAGC-AGCACTCTACC-AC
gi|164345171+        ATTGG-ATNTTCTGGGTGGGACCATTCATTGGT-GCAGCACTAGC-AGCACTCTACC-AC
gi|84144380+         ATTGG-ATTTTCTGGGTGGGACCATTCATTGGT-GCAGCACTAGC-AGCACTCTACCCAC
gi|164255409+        ATTGG-ATTTTCTGGGTGGGACCATTCATTGGT-GCAGCACTAGC-AGCACTCTACC-AC
gi|164293662+        ATTGG-ATTTTCTGGGTGGGACCATTCATTGGT-GCAGCACTAGC-AGCACTCTACC-AC
gi|109825953+        ATTGG-ATTTTCTGGGTGGGACCATTCATTGGT-GCAGCACTAGC-AGCACTCTACC-AC
gi|84174539-         ATTGG-ATTTTCTGGGTGGGACCATTCATTGGT-GCAGCACTAGC-AGCACTCTACC-AC
gi|109842739+        ATTGG-ATTTTCTGGGTGGGACCATTCATTGGT-GCAGCACTAGC-AGCACTCTACC-AC
                     ____________________________________________________________
consensus            ATTGG-ATTTTCTGGGTGGGACCATTCATTGGT-GCAGCACTAGC-AGCACTCTACC-AC
                     H  W   I  F  W  V  G  P  F  I  G   A  A  L  A   A  L  Y   H

                          .   :   .   :   .   :   .   :   .   :   .   :
gi|164338038+        GTTGGTTGTGATCAGAGCCCTTCCTT-TCAAAACCAAGTGA
gi|78339752+         GTTG-TTGTGATCAGAGCCATTCCTT-TCAAATC-AAGTGA
gi|118132685+        GTTG-TTGTGATCAGAGCCATTCCTT-TCAAATCAAAGTGA
gi|82755095+         GTTG-TTGTGATCAGAGCCATTCCTT-TCAAATCAAAGTGA
gi|84144149+         GTTG-TTGTGATCAGAGCCATTCCTT-TCAAATCAAAGTGA
gi|164345171+        GTTG-TTGTGATCAGAGCCATTCCTGNTTCAATC-AAGTGA
gi|84144380+         GTTG-TTGTGATCAGAGCCATTCCTT
gi|164255409+        GTTG-TTGTGATCAGAGCCATCCCTT-TCAACTCAACGTGA
gi|164293662+        GTTG-TTGTGATCAGAGCCATTCCTT-TCAAATCAAAGTGA
gi|109825953+        GTTG-TTGTGATCAGAGCCATTCCTT-TCAAATTNAAGTGA
gi|84174539-         GTTG-TTGTGATCAGAGCCATTCCTT-TCAAATCAAAGTGA
gi|109842739+        GTTG-TTGTGATCAGAGCCATTCCTT-TCGAATCAAAGTGA
                     ____________________________________________________________
consensus            GTTG-TTGTGATCAGAGCCATTCCTT-TCAAATCAAAGTGA
                      V   V  V  I  R  A  I  P   F  K  S  K  -
```
